# Mental ill-health during COVID-19 confinement

**DOI:** 10.1186/s12888-021-03191-5

**Published:** 2021-04-14

**Authors:** Eva Jané-Llopis, Peter Anderson, Lidia Segura, Edurne Zabaleta, Regina Muñoz, Gemma Ruiz, Jürgen Rehm, Carmen Cabezas, Joan Colom

**Affiliations:** 1 Ramon Llull University, ESADE Business School, Barcelona, Spain; 2 CAPHRI Care and Public Health Research Institute, Maastricht University, POB 616, Maastricht, MD 6200 The Netherlands; 3 Institute for Mental Health Policy Research, CAMH, 33 Russell Street, Toronto, ON M5S 2S1 Canada; 4 Population Health Sciences Institute, Newcastle University, Baddiley-Clark Building, Richardson Road, Newcastle upon Tyne, NE2 4AX UK; 5 Public Health Agency of Catalonia, Department of Health, Government of Catalonia, Programme on Substance Abuse, Roc Boronat 81-95, 08005 Barcelona, Spain; 6 Fundació Institut Universitari per a la recerca a l’Atenció Primària de Salut Jordi Gol i Gurina (IDIAPJGol), Gran Via de les Corts Catalanes 587, 08007 Barcelona, Spain; 7 Gerència Territorial de Barcelona, Institut Català de la Salut, Balmes 22, 08007 Barcelona, Spain; 8 Nursing Department, Nursing Faculty, Universitat de Girona, Emili Grahit 77, 17003 Girona, Spain; 9 Universitat Autònoma de Barcelona, Bellaterra, 08193 Cerdanyola del Vallès, Spain; 10 Dalla Lana School of Public Health & Department of Psychiatry, University of Toronto, 6th Floor, 155 College Street, Toronto, Ontario M5T 3M7 Canada; 11 Institute of Clinical Psychology and Psychotherapy & Center for Clinical Epidemiology and Longitudinal Studies, Technische Universität Dresden, Chemnitzer Str. 46, D-01187 Dresden, Germany; 12 Department of International Health Projects, Institute for Leadership and Health Management, I.M. Sechenov First Moscow State Medical University, Trubetskaya str., 8, b. 2, Moscow, Russian Federation 119992

**Keywords:** COVID-19, Confinement, Mental ill-health, Addictions

## Abstract

**Background:**

Confinement due to COVID-19 has increased mental ill-health. Few studies unpack the risk and protective factors associated with mental ill-health and addictions that might inform future preparedness.

**Methods:**

Cross-sectional on-line survey with 37,810 Catalan residents aged 16+ years from 21 April to 20 May 2020 reporting prevalence of mental ill-health and substance use and associated coping strategies and behaviours.

**Results:**

Weighted prevalence of reported depression, anxiety and lack of mental well-being was, respectively, 23, 26, and 75%, each three-fold higher than before confinement. The use of prescribed hypnosedatives was two-fold and of non-prescribed hypnosedatives ten-fold higher than in 2018. Women, younger adults and students were considerably more likely, and older and retired people considerably less likely to report mental ill-health. High levels of social support, dedicating time to oneself, following a routine, and undertaking relaxing activities were associated with half the likelihood of reported mental ill-health. Worrying about problems living at home, the uncertainty of when normality would return, and job loss were associated with more than one and a half times the likelihood of mental ill-health. With the possible exception of moderately severe and severe depression, length of confinement had no association with reported mental ill-health.

**Conclusions:**

The trebling of psychiatric symptomatology might lead to either to under-identification of cases and treatment gap, or a saturation of mental health services if these are not matched with prevalence increases. Special attention is needed for the younger adult population. In the presence of potential new confinement, improved mental health literacy of evidence-based coping strategies and resilience building are urgently needed to mitigate mental ill-health.

**Supplementary Information:**

The online version contains supplementary material available at 10.1186/s12888-021-03191-5.

## Background

At the beginning of the COVID-19 pandemic, the World Health Organization alerted about the serious consequences of COVID-19 confinements on mental ill-health and addictions such as potential high-risk drinking [1]. At early stages of the pandemic, individual studies in China showed that one half the general population in confinement suffered moderate-to-severe negative psychological impact, and one third moderate-to-severe anxiety [2]. Similarly, a little later, a study with 7236 respondents found a 35.1% prevalence of anxiety and a 20.1% prevalence of depressive symptoms [3]. A later meta-analysis of both the general population and health care providers with 162,639 respondents indicated an average prevalence during the beginning of confinement of 33% for anxiety and of 28% for depression across 62 studies [4]. Although with less clear output, early attention was also been drawn to possible increases in tobacco and alcohol use [5], with one Chinese study reporting increases in hazardous alcohol drinking [6], and others reporting decreases in alcohol and tobacco consumption [7, 8].

More broadly, systematic reviews, including European studies, albeit largely of relatively small sample sizes, using symptom-based scales, have found during COVID-19 confinement prevalence rates of depression of 25% (95% confidence intervals, 18–33%) [9], and prevalence rates of anxiety also of 25% (95% confidence intervals, 21–29%) [10].

Few studies have set out to investigate the impact of length of confinement and whether the number of weeks at home increases levels of symptomatology or severity. Similarly, there has been less dedicated attention to the factors that mitigate worsening of mental ill-health during confinement and those that seem to aggravate symptomatology. This is essential to understand what should be included in tailored prevention interventions to be delivered under pandemic conditions, namely what is protective overall, what each vulnerable group can benefit from, and what structural risk factors need to be most mitigated [11].

This cross-sectional study’s objective is to addresses a knowledge gap by reporting: the impact of confinement on mental ill-health and addictions for a study population already more than six to ten weeks into confinement; the link between reported length of confinement and severity; and, detailed unpacking of the associated risk and protective factors that can provide insight for intervention preparedness in view of forthcoming partial or total confinements.

## Methods

### Situation

National confinement was implemented in Spain on 14th March 2020. Citizens were confined to their homes with the only exceptions of going outside to undertake essential activities, namely acquiring food and medicine, in case of emergency, or if they were performing essential work. The de-confinement plan was announced on 28th April, and limitations were reduced over a staggered period between 28th May and 21st June. For non-essential workers, full confinement in Spain lasted ten weeks, not even being allowed to go outside to exercise, until the first de-confinement measures were put in place. Catalonia, an autonomous region in Spain with a population 7.7 million [12] in which the survey was implemented, was the one of the regions most severely hit by COVID-19.

### Study design and participants

A voluntary cross-sectional web-based population survey was launched on 21st April 2020 in Catalonia, 5 weeks after confinement had been imposed, using a snowball dissemination strategy through social media and government official channels, including government information websites and health and education settings. The survey was completed by 40,185 people over 30 days, terminating on 20th May 2020, 1 week prior to the first phase of relaxation of confinement. Since not all workers were confined for the full ten-week duration of confinement, the length of time reported in confinement by respondents does not necessarily correlate with the date that the survey was completed. Following inclusion criteria, the final sample comprised 37,810 residents of Catalonia aged 16 or more years. Respondents with a non-Catalan postcode (2322 respondents) or who gave a stated age under 16 years of age (53 respondents) were excluded. At the time of the beginning of data collection there were 9509 deaths due to COVID-19 in Catalonia, and 11,879 by 20th May when data collection finished.

The study was approved by IDIAPJGol’s Ethics Committee (code 20/079-PVC) and followed international regulations, including the Declaration of Helsinki and the Code of Ethics. All personal data was handled following Regulation (EU) 2016/679 and the National Organic Law 3/2018 on the Protection of Personal Data. Participation consent was provided when answering the survey which was voluntary and anonymous. Data was stored on secure servers and limited to the study purposes.

### Survey construction and measures

The survey, available in Catalan and Spanish, included globally validated instruments used in the Spanish National Health Survey [13], including the alcohol and drugs survey [14], and in the Catalan Health Survey [15]. In addition, the survey included newly created COVID-19 questions related to confinement, piloted, and iterated in two phases.

#### Psychological and health behaviour measures

Depression was measured with the Patient Health Questionnaire 8 rather than the Patient Health Questionnaire 9 to match the Catalan Health Survey data [15], with a cut-off point of 10 or above to indicate likelihood of depressive disorder overall, and for severity, above 15 to indicate moderate to severe depression and above 20 to indicate severe depression [16]. Anxiety was measured with the Generalized Anxiety Disorder Scale-7 using the standard cut of point of 10 or above for likelihood of overall anxiety disorder and for severity above 15 for severe anxiety [17]. Emotional well-being was assessed with the Short Warwick–Edinburgh Mental Well-being Scale [18], with a cut-off point under 26 indicating lack of mental well-being as for the Catalan Health Survey [15]. Alcohol use was measured with the Alcohol Use Disorders Identification Test, three alcohol consumption questions (AUDIT-C), with a score of 5 and above as indicative of higher-risk drinking [19]. The European Model Questionnaire was used for tobacco use, frequency of use of non-prescribed and prescribed hypnosedatives (tranquilizers, sedatives or sleeping pills), and use of drugs (cannabis, marihuana or hashish) [20]. Social support was measured by the Oslo Social Support Scale [21].

#### Mitigating and aggravating (risk and protective) factors

A new set of confinement and pandemic specific variables were developed, based on COVID-19 studies [3, 6, 11], ongoing recommendations to help mental health in confinement [1, 22], and known protective and risk factors for mental health [23]. After two rounds, the final list of variables included living condition variables (e.g., number of rooms in household, availability of garden or balcony), a set of key coping strategies (e.g., undertaking relaxing activities, following a routine), risk factors (e.g., being a frontline worker, having family members in hospital or deceased because of COVID-19), and related individual, social and economic concerns (e.g., worried about problems at home, the uncertainty of the situation, economic concerns). Supplement Table 1 lists the included risk and protective variables, with their definitions, cut-off scores, and distributions in the sample population.

### Statistical analyses

The main dependent variables were dichotomized as present or absent for reported depression, anxiety, lack of mental well-being, positive AUDIT-C score, use of non-prescribed and prescribed hypnosedatives, being a smoker, reported smoking more during confinement if a smoker, and use of cannabis. Socio-demographic characteristics of the sample were dummy coded present or absent for gender (female, male, other) age (16–44 years, 45–64 years, 65+ years), final level of education obtained (primary, secondary, university, postgraduate) and occupation (health professional, other frontline worker, other worker, on sick leave, unemployed, student, other, retired). Means and 95% confidence intervals (estimated with bootstrapping, *n* = 1000), and odds ratios with 95% confidence intervals (estimated with general linear models, using binomial distribution, and controlling for gender, age and educational level as appropriate) are reported for the dependent variables by the sociodemographic characteristics. When reporting the overall prevalence of mental ill-health and substance use identified by our survey, the results were weighted separately, but sequentially by distributions of gender (male or female), age (16–44, 45–64, and 65+ years), and educational level (primary, secondary, and university or higher) as reported by government statistics of the Catalan population [12].

The dependent variables were aggregated as means per each day across the 30 days of the survey, and per each week of the number of weeks reported in confinement. Linear regression analyses were undertaken separately for each of the dependent variables, with, respectively, sequential days of the survey, and the number of weeks reported in confinement as independent variables to examine reported changes over time; Durbin-Watson tests identified no autocorrelation.

Forty seven potential risk and protective factors and twelve dummy socio-demographic variables (independent variables, Supplement Table 1) were entered into general linear models, using binomial distribution, to estimate odds ratios with 95% confidence intervals for the presence of the three main dependent variables, depression, anxiety and lack of mental well-being. Given the large number of independent variables, for the main results, we extracted variables that had an odds ratio of either greater than 1.5 or of less than 0.67, with *p* value of less than 0.001 that were present for at least one condition (depression, anxiety and lack of mental well-being) and for at least one demographic group (gender or age groups). We undertook a path analysis of the direct and indirect associations via reported mental ill-health between the extracted variables and reported heavy drinking and substance use.

We undertook categorical principal components analyses to identify two groupings of a reduced number of potential predictor variables from the original full set described in Supplement Table 1. After the first run, variables that had total vector coordinates of less than 0.5 were removed for the second run; and, after the second run, variables that had total vector coordinates of less than 1.0 were removed for the third run. Plots of dimension loadings are reported for the third run. We created a scale from the results of the categorical principal components analysis of the number of items of worry a respondent reported (from 0 to 10) and the number of reported protective behaviours to reduce the risk of mental ill-health (from 0 to 3).

## Results

### Demographic characteristics

Compared with the Catalan population [12], the sample of 37,810 respondents included a higher proportion of women, the age group 45–64 years, and respondents with higher levels of achieved education, Supplement Table 2. Of the 23,043 employed respondents in the sample, 4503 described themselves as a health professional (11.9% of the total sample) and 4393 (11.6% of whole sample) as other frontline worker (e.g., those who had to work during the confinement, such as supermarket workers, police, public transport workers).

### Depression, anxiety, addictions, and mental well-being before and during COVID-19 confinement

Supplement Tables 3a to 3c display the demographic characteristics and percentages of the sample with reported depression, anxiety, lack of mental well-being (Supplement Table 3a), substance use (Supplement Table 3b), and consumption of non-prescribed and prescribed hypnosedatives (Supplement Table 3c). Linear regression found that the reported percentages for all dependent variables did not change over the 30 days during which the survey was completed (Supplement Table 3a-3c, last rows, titled ‘Regression, Durbin Watson’). Of the 7598 respondents who reported depression (23.8% of the total sample), 4450 reported moderate (14.0% of the total sample), 2159 moderately severe (6.8%) and 989 (3.1%) severe depression. Of the 8783 respondents who reported anxiety (27.3% of the total sample), 5348 reported moderate (16.6% of the total sample), and 3435 (10.7%) severe anxiety. The reported percentages of those with moderately severe (coefficient = 0.054 (95% CI = -0.04 to 0.15) and severe (coefficient = 0.030 (95% CI = -0.05 to 0.11) depression and with severe anxiety (coefficient = 0.092 (95% CI = -0.07 to 0.27) did not change over the 30 days during which the survey was completed.

Table 1 displays the reported prevalence of mental ill-health and substance use before and during COVID-19 confinement, with results from the sample during COVID-19 confinement weighted by gender, age and achieved educational level of the population distribution of Catalonia [12]. The reported prevalence of depression during confinement, 22.8%, was three times higher than the prevalence during 2018, 7.6% [15]. Similarly, the prevalence of anxiety was more than three times higher, and lack of mental well-being just under three times higher [13, 15].

**Table 1:** Prevalence of mental ill health and substance use before and during COVID-19 confinement

	CATALONIA before COVID		CATALONIA during COVID-19 Confinement^**a**^	
	n	%	N	%
Depression	3551	7.6% ^c^	31,873	22.8%
Anxiety^b^	2621	6.7 ^b^	32,185	26.9%
Lack of mental well-being	3536	26.2% ^c^	37,596	74.8%
Higher risk drinking	1529	8.6% ^d^	37,261	9.1%
Smoking	3557	25.6% ^c^	37,820	23.0%
Cannabis / hashish (in last 30 days)	2044	11.9% ^d^	37,820	3.7%
Prescribed hypnosedatives	2043	5.9% ^d^	37,820	17.7%
Non-prescribed hypnosedatives	2044	0.6% ^d^	37,820	6.5.%

^a^Weighted separately, but sequentially by distributions of gender (male or female), age (16–44, 45–64, and 65+ years), and educational level (primary, secondary, and university or higher) as reported by government statistics of the Catalan population [12]

^b^Anxiety was measured by Generalized Anxiety Disorder Scale-7. There is no previous data from Catalonia with this instrument. It is compared with results of the Spanish National Health Survey (2017) [13], using a different instrument which reported a prevalence of 6.7% for chronic anxiety; the same survey found a prevalence of 6.7% for depression in the Spanish population

^c^Catalan Health Survey 2018 [15]

^d^Spanish Alcohol and Drug Survey 2017 [14], Catalonia only data. For drugs (hypnosedatives and cannabis) the prevalence estimates are based on % of people who reported having taken drugs in the last 30 days. For hypnosedatives with prescription, the data before COVID-19 also includes without prescription

The reported prevalence of higher risk drinking and smoking were marginally lower, Table 1, although 40% of smokers in the sample reported that they smoked more during confinement than before (Supplement Table 3b) [12]. The reported use of cannabis was a third less [13]. The use of prescribed hypnosedatives was three times higher, and for non-prescribed hypnosedatives ten times higher.

### Most and less affected groups

Supplement Table 3a to 3c displays the odds ratios for the dependent variables by the socio-demographic characteristics. The most affected groups were those aged 16–44 years, women, those with a primary level of education, and students. Of the younger adults, the prevalence of reported mental ill-health was higher the younger the age group, Table 2.

**Table 2:** Reported prevalence (%), with 95% CI from bootstrapping (*n* = 1000), of mental ill-health by age group for those aged 16–44 years

Age	Depression	Anxiety	Lack of mental well-being
**15–24**	42.49 (40.29 to 44.52)	37.31 (35.14 to 39.46)	90.93 (89.65 to 92.19)
**25–34**	29.46 (28.05 to 31.01)	30.30 (28.84 to 31.66)	87.40 (86.43 to 88.39)
**35–44**	21.71 (20.81 to 22.59)	28.49 (27.56 to 29.47)	83.39 (82.59 to 84.21)

The least affected groups were those aged 65 or more years and the retired. Compared with all other employment groups, health professionals were 27% more likely to report anxiety, but were no more likely to report depression or lack of mental well-being.

### Risk factors for mental health

Supplement Table 4 displays the results of the associations between all risk and protective factors and reported depression, (Supplement Table 4a), anxiety, (Supplement Table 4b) and lack of mental well-being (Supplement Table 4c). Table 3 displays the top risk factors, and Table 4 the top protective factors for depression, anxiety and lack of mental well-being, as defined in statistical analyses section. In Table 3, odds ratios underlined in bold are those greater than 1.5, with probability values for confidence intervals of less than 0.001; odds ratios in bold are those greater than 1.25, with probability values for confidence intervals of less than 0.001; odds ratios that are not bold or underlined are not greater than 1.25, or have a probability value of 0.001 or greater. In Table 4, odds ratios underlined in bold are those less than 0.67, with probability values for confidence intervals of less than 0.001; odds ratios in bold are those less than 0.80, with probability values for confidence intervals of less than 0.001; odds ratios that are not bold or underlined are not less than 0.80, or have a probability value of 0.001 or greater.

**Table 3:** Top risk factors associated with depression, lack of mental well-being and anxiety^a^

Risk factors	Depression						Anxiety						Lack of mental well-being					
	TOTAL	women	men	16–44	45–64	65+	TOTAL	women	men	16–44	45–64	65+	TOTAL	women	men	16–44	45–64	65+
Worry about problems due living together at home^b^	**1.80 (1.63 to 1.99)**	**1.70 (1.52–1.91)**	**2.29 (1.81–2.89**	**1.65 (143–1.90)**	**1.81 (1.55–2.11)**	**2.99 (1.87–4.80)**	**2.05 (1.86 to 2.27)**	**1.95 (1.74 to 2.18)**	**2.58 (2.07 to 3.20)**	**2.08 (1.80 to 2.40)**	**1.75 (1.51 to 2.03)**	**6.06 (3.84 to 9.55)**	**1.93 (1.64–2.27)**	**2.20 (1.78 to 2.72)**	**1.56 (1.20 to 2.03)**	**2.11 (1.56 to 2.85)**	**1.81 (1.46 to 2.24)**	1.84 (1.13 to 3.01)
Worry about the uncertainty of when and how normality will return^b^	**1.59 (1.49 to 1.70)**	**1.62 (1.51 to 1.74)**	**1.58 (1.36 to 1.83)**	**1.59 (1.45 to 1.74)**	**1.52 (1.38 to 1.68)**	**2.29 (1.75 to 3.01)**	**1.89 (1.78 to 2.01)**	**1.87 (1.75 to 2.00)**	**2.06 (1.79 to 2.36)**	**1.90 (1.73 to 2.07)**	**1.81 (1.65 to 1.98)**	**2.38 (1.88 to 3.02)**	**1.69 (1.58 to 1.81)**	**1.79 (1.65–1.95)**	**1.49 (1.32 to 1.68)**	**1.91 (1.68 to 2.18)**	**1.56 (1.43 to 1.71)**	**1.79 (1.51 to 2.11)**
Suffer from chronic diseases that increase own risk of COVID-19 infection^c^	**1.56 (1.44 to 1.68)**	**1.58 (1.44 to 1.72)**	**1.56 (1.31 to 1.85)**	**1.49 (1.30 to 1.70)**	**1.55 (1.40 to 1.73)**	**2.02 (1.57 to 2.61**)	**1.36 (1.26 to 1.47)**	**1.33 (1.22 to 1.46)**	**1.50 (1.28 to 1.76)**	**1.36 (1.19 to 1.55)**	**1.35 (1.22 to 1.50)**	1.43 (1.13 to 1.82)	**1.28 (1.18 to 1.40)**	**1.26 (1.13 to 1.40)**	**1.37 (1.19 to 1.57)**	1.23 (1.01 to 1.49)	**1.25 (1.12 to 1.39)**	**1.47 (1.23 to 1.74)**
Worry about uncertain future of own job^b^	**1.60 (1.49–1.72)**	**1.58 (1.45 to 1.71)**	**1.78 (1.51 to 2.09)**	**1.43 (1.29 to 1.59)**	**1.80 (1.61 to 2.01)**	1.65 (1.13 to 2.41)	**1.49 (1.39 to 1.60)**	**1.49 (1.38 to 1.62)**	**1.54 (1.32 to 1.80)**	**1.33 (1.20 to 1.48)**	**1.66 (1.50 to 1.85)**	1.48 (1.04 to 2.10)	**1.54 (1.41 to 1.68)**	**1.47 (1.32 to 1.64)**	**1.70 (1.45 to 1.98)**	**1.58 (1.36 to 1.84)**	**1.56 (1.38 to 1.76)**	1.38 (1.03 to 1.84)
Worry about the COVID-19’s negative economic consequences^b^	**1.31 (1.23 to 1.41)**	**1.32 (1.22 to 1.42)**	1.28 (1.08 to 1.50)	**1.31 (1.18 to 1.45)**	1.24 (1.13 to 1.37)	**2.02 (1.56 to 2.61)**	**1.46 (1.37 to 1.56)**	**1.49 (1.38 to 1.60)**	**1.29 (1.11 to 1.50)**	**1.40 (1.27 to 1.55)**	**1.50 (1.37 to 1.64)**	**1.49 (1.18 to 1.88)**	**0.88 (0.82 to 0.94)**	0.88 (0.81 to 0.96)	0.87 (0.76 to 0.99)	**0.77 (0.67 to 0.89)**	0.92 (0.84 to 1.01)	0.92 (0.77 to 1.11)
Worry about not being able to go out of the house or visit loved ones^b^	**1.39 (1.31 to 1.48)**	**1.38 (1.29 to 1.48)**	**1.44 (1.24 to 1.67)**	**1.31 (1.20 to 1.44)**	**1.43 (1.30 to 1.58)**	**1.65 (1.28 to 2.15)**	**1.38 (1.30 to 1.46)**	**1.36 (1.27 to 1.46)**	**1.42 (1.24 to 1.63)**	**1.37 (1.25 to 1.50)**	**1.34 (1.23 to 1.47)**	**1.58 (1.26 to 1.98)**	**1.30 (1.21 to 1.39)**	**1.35 (1.24 to 1.46)**	1.18 (1.04 to 1.33)	**1.46 (1.29 to 1.66)**	1.23 (1.13 to 1.35)	1.22 (1.03 to 1.45)
Worry about anxious children who do not know what to do, leading to tensions and bad child behaviour^b^	**1.45 (1.33 to 1.58)**	**1.48 (1.34 to 1.62)**	1.30 (1.05 to 1.61)	**1.47 (1.29 to 1.68)**	**1.48 (1.30 to 1.67)**	1.53 (1.11 to 2.09)	**1.62 (1.49 to 1.76)**	**1.61 (1.47 to 1.76)**	**1.72 (1.43 to 2.08)**	**1.57 (1.39 to 1.78)**	**1.68 (1.49 to 1.88)**	**1.90 (1.44 to 2.51)**	**1.44 (1.29 to 1.60)**	**1.50 (1.32 to 1.71)**	1.29 (1.07 to 1.56)	**1.42 (1.17 to 1.73)**	**1.56 (1.35 to 1.80)**	1.25 (0.97 to 1.61)
Being responsible for dependent people that take up most of the time^c^	1.27 (1.01 to 1.60)	1.38 (1.08 to 1.76)	0.71 (0.34 to 1.45)	0.78 (0.49 to 1.24)	1.43 (1.08 to 1.90)	1.83 (0.86 to 3.91)	**1.61 (1.30 to 2.00)**	**1.61 (1.27 to 2.03)**	1.37 (0.75 to 2.49)	1.38 (0.89 to 2.14)	**1.70 (1.30 to 2.22)**	1.59 (0.78 to 3.24)	0.92 (0.71 to 1.18)	0.90 (0.68 to 1.19)	0.92 (0.51 to 1.65)	0.65 (0.36 to 1.15)	0.92 (0.67 to 1.26)	1.40 (0.77 to 2.54)
Worry about oneself or family member being infected by COVID-19^b^	1.17 (1.10 to 1.24)	1.17 (1.09 to 1.25)	1.21 (1.04 to 1.40)	1.12 (1.03 to 1.23)	1.20 (1.09 to 1.31)	1.41 (1.07 to 1.84)	**1.49 (1.40 to 1.58)**	**1.51 (1.41 to 1.61)**	**1.44 (1.26 to 1.65)**	**1.42 (1.31 to 1.55)**	**1.55 (1.41 to 1.69)**	**1.66 (1.29 to 2.12)**	1.16 (1.10 to 1.24)	1.17 (1.09 to 1.26)	1.15 (1.03 to 1.28)	**1.27 (1.13 to 1.42)**	1.12 (1.03 to 1.21)	1.22 (1.05 to 1.42)
Worry about being alone and not being able to take care of oneself^b^	1.17 (1.07 to 1.28)	1.21 (1.10 to 1.33)	1.01 (0.81 to 1.25)	1.16 (1.01 to 1.33)	1.16 (1.03 to 1.32)	1.25 (0.94 to 1.68)	1.22 (1.12 to 1.32)	**1.25 (1.14 to 1.37)**	1.14 (0.93 to 1.38)	**1.30 (1.14 to 1.50)**	1.10 (0.98 to 1.23)	**1.56 (1.21 to 2.02)**	1.11 (1.01 to 1.23)	1.14 (1.01 to 1.28)	1.07 (0.89 to 1.27)	1.21 (0.96 to 1.52)	1.04 (0.91 to 1.18)	1.29 (1.04 to 1.61)
Spending more than 2 h a day reading COVID-19 news or information^c^	1.18 (1.09 to 1.27)	1.16 (1.07 to 1.26)	1.23 (1.05 to 1.44)	1.10 (0.98 to 1.24)	1.22 (1.10 to 1.35)	1.32 (1.02 to 1.72)	**1.32 (1.23 to 1.41)**	**1.30 (1.20 to 1.41)**	**1.36 (1.17 to 1.57)**	**1.33 (1.19 to 1.49)**	**1.27 (1.15 to 1.40)**	**1.57 (1.25–1.98)**	1.16 (1.08 to 1.25)	1.12 (1.02 to 1.22)	1.25 (1.10 to 1.42)	1.03 (0.88 to 1.21)	1.24 (1.12 to 1.37)	1.09 (0.92 to 1.28)

^a^Odds ratios underlined in bold are those greater than 1.5, with probability values for confidence intervals of less than 0.001; odds ratios in bold are those greater than 1.25, with probability values for confidence intervals of less than 0.001; odds ratios that are not underlined or bold are not greater than 1.25, or have a probability value of 0.001 or greater

^b^Worry a lot (score 4 on a 4-point Likert scale from 1, not worried to 4, worried a lot) versus not score 4

^c^Present versus absent

**Table 4:** Top protective factors associated with depression, lack of mental well-being and anxiety^a^

Protective Factors & coping behaviours	Depression						Anxiety						Lack of mental well-being					
	TOTAL	women	men	16–44	45–64	65+	TOTAL	women	men	16–44	45–64	65+	TOTAL	women	men	16–44	45–64	65+
Following a routine^b^	**0.58 (0.55 to 0.62)**	**0.60 (0.56 to 0.64)**	**0.52 (0.45 to 0.59)**	**0.59 (0.54 to 0.65)**	**0.56 (0.51 to 0.61)**	0.70 (0.55 to 0.89)	**0.80 (0.75 to 0.85)**	0.82 (0.77 to 0.87)	**0.73 (0.64 to 0.83)**	**0.79 (0.72 to 0.86)**	0.82 (0.75 to 0.89)	0.79 (0.63 to 0.98)	**0.58 (0.54 to 0.61)**	**0.54 (0.50 to 0.59)**	**0.63 (0.57 to 0.70)**	**0.52 (0.46 to 0.59)**	**0.57 (0.52 to 0.62)**	**0.70 (0.60 to 0.81)**
High social support v low social support (3-point scale)	**0.54 (0.50 to 0.59)**	**0.56 (0.51 to 0.61)**	**0.47 (0.38 to 0.57)**	**0.55 (0.49 to 0.62)**	**0.54 (0.47 to 0.60)**	0.56 (0.40 to 0.80)	**0.65 (0.60 to 0.71)**	**0.68 (0.62 to 0.74)**	**0.57 (0.48 to 0.69)**	**0.64 (0.57 to 0.72)**	**0.65 (0.58 to 0.73)**	0.75 (0.55 to 1.03)	**0.57 (0.52 to 0.62)**	**0.59 (0.53 to 0.65)**	**0.54 (0.46 to 0.62)**	**0.55 (0.47 to 0.64)**	**0.60 (0.54 to 0.68)**	**0.50 (0.41 to 0.62)**
Dedicating time to oneself^b^	**0.60 (0.54 to 0.67)**	**0.61 (0.55 to 0.69)**	**0.57 (0.44 to 0.73)**	**0.68 (0.59 to 0.80)**	**0.54 (0.46 to 0.63)**	**0.52 (0.37 to 0.72)**	**0.69 (0.63 to 0.76)**	**0.71 (0.64 to 0.78)**	**0.62 (0.50 to 0.78)**	**0.68 (0.59 to 0.79)**	**0.65 (0.57 to 0.75)**	0.80 (0.61 to 1.04)	**0.58 (0.54 to 0.62)**	**0.57 (0.52 to 0.61)**	**0.61 (0.54 to 0.70)**	**0.58 (0.50 to 0.66)**	**0.57 (0.52 to 0.63)**	**0.57 (0.49 to 0.67)**
Not eating more to cope with the situation^c^	**0.63 (0.59 to 0.67)**	**0.63 (0.59 to 0.68)**	**0.61 (0.52 to 0.73)**	**0.66 (0.60 to 0.73)**	**0.61 (0.55 to 0.67)**	**0.63 (0.49 to 0.80)**	0.88 (0.83 to 0.93)	0.88 (0.82 to 0.94)	0.89 (0.78 to 1.01)	0.89 (0.81 to 0.97)	0.88 (0.81 to 0.97)	0.80 (0.65 to 1.00)	**0.78 (0.73 to 0.82)**	**0.77 (0.72 to 0.82)**	**0.80 (0.73 to 0.89)**	**0.80 (0.72 to 0.89)**	**0.76 (0.70 to 0.82)**	0.82 (0.71 to 0.94)
Spending more time doing activities with the family^b^	**0.70 (0.65 to 0.76)**	**0.72 (0.67 to 0.78)**	**0.61 (0.53 to 0.71)**	**0.74 (0.66 to 0.81)**	**0.71 (0.63 to 0.79)**	**0.65 (0.47 to 0.90)**	**0.73 (0.68 to 0.78)**	**0.74 (0.69 to 0.80)**	**0.70 (0.60 to 0.82)**	**0.75 (0.68 to 0.82)**	**0.72 (0.65 to 0.80)**	0.81 (0.63 to 1.06)	**0.69 (0.65 to 0.73)**	**0.69 (0.64 to 0.74)**	**0.70 (0.63 to 0.78)**	**0.67 (0.60 to 0.75)**	**0.68 (0.63 to 0.74)**	0.82 (0.70 to 0.95)
Undertaking relaxing activites (e.g. listening to music)^b^	**0.78 (0.73 to 0.84)**	**0.76 (0.70 to 0.82)**	0.88 (0.75 to 1.02)	0.82 (0.74 to 0.91)	**0.74 (0.66 to 0.82)**	0.71 (0.54 to 0.93)	**0.72 (0.68 to 0.77)**	**0.72 (0.66 to 0.77)**	**0.74 (0.63 to 0.85)**	**0.73 (0.66 to 0.81)**	**0.73 (0.66 to 0.81)**	**0.63 (0.49 to 0.79)**	**0.71 (0.67 to 0.76)**	**0.70 (0.65 to 0.75)**	**0.75 (0.67 to 0.83)**	**0.68 (0.61 to 0.76)**	**0.71 (0.65 to 0.77)**	**0.74 (0.64 to 0.86)**
Having children whose care takes half of the time or less^d^	**0.76 (0.70 to 0.83)**	**0.76 (0.70 to 0.84)**	0.75 (0.62 to 0.91)	**0.53 (0.46 to 0.61)**	0.94 (0.84 to 1.05)	1.84 (0.84 to 4.04)	1.00 (0.93 to 1.09)	0.97 (0.89 to 1.06)	1.16 (0.98 to 1.37)	0.89 (0.78 to 1.02)	1.09 (0.98 to 1.20)	1.92 (0.92 to 3.99)	1.03 (0.95 to 1.11)	1.00 (0.91 to 1.09)	1.10 (0.96 to 1.25)	**0.74 (0.64 to 0.86)**	1.14 (1.05 to 1.25)	1.61 (0.89 to 2.88)

^a^Odds ratios underlined in bold are those less than 0.67, with probability values for confidence intervals of less than 0.001; odds ratios in bold are those less than 0.80, with probability values for confidence intervals of less than 0.001; odds ratios that are not underlined or bold are not less than 0.80, or have a probability value of 0.001 or greater

^b^Almost every day (score 4 on a 4-point Likert scale from 1, not any day to 4, almost every day) versus not score 4

^c^Not any day (score 1 on a 4-point Likert scale from 1, not any day to 4, almost every day) versus not score 1

^d^Present versus absent

For all ages and gender groups, the associated likelihood of reporting depression, anxiety, and lack of mental well-being was doubled for those who worried very much about “difficulties living together at home”, “uncertainty about how or when normal life will be resumed”, and “suffering from chronic diseases that predispose to a higher risk of COVID-19”. In general all risk factors had higher odds ratios (i.e., more at risk associations) as reported severity of depression or anxiety was greater (Supplement Table 5).

For gender and age group-specific findings, worrying about “one’s future work will get worse” for those aged 45–65 years, and “not being able to go out or visit people important to them” for those aged 65+ years, were associated with an one and a half times increase in the likelihood of reported depression, anxiety, and lack of mental well-being. An increased one and a half time likelihood of anxiety was found for: both men and women who reported being “very concerned that their children were restless, did not know what to do and expressed behavioural problems”; women aged 45–64 years “with dependents whose care takes up almost all of their time”; adults over the age of 44 years who “were very concerned about themselves or their family getting COVID-19”; and, adults aged 65+ years, “if they spent more than two hours a day consulting COVID-19 news” or if they “were very worried because they were alone and unable to take care of themselves”.

### Coping mechanisms and protective factors and behaviours

Protective factors associated with half the likelihood of suffering from depression, anxiety and lack of mental well-being included: “having high social support”, “dedicating time to oneself almost every day”, and, “being older”, in particular being aged 65+ years. Behaviours associated with half the likelihood of reporting depression and lack of mental well-being included: “following a routine almost every day”; “dedicating time to oneself (personal image, taking care of hair etc) almost every day”; and, “not eating more to help cope with the situation”. In general all protective factors and coping behaviours had lower odds ratios (i.e., more protective associations) as reported severity of depression or anxiety was greater (Supplement Table 5).

For gender and age group-specific findings, the likelihood of reporting depression was halved in men who “spent more time doing activities with the family” and younger adults “who had children that only occupied half of the time or less”. “Undertaking relaxing activities (e.g., listening to music)” reduced the associated likelihood of reporting anxiety for both younger adults (aged 16–44 years), and for older adults, aged 65+ years.

### Living conditions and time in confinement did not affect mental health

Respondents’ living conditions (having a balcony, terrace or garden; number of rooms in the house; and number of people living in the house) had no associations with reported levels of depression, anxiety or lack of mental well-being. For the population as a whole, the number of reported weeks in confinement was not associated with reported mental ill-health. One exception to this was men for whom, comparing nine or more weeks to less than 9 weeks in confinement, were 60% more likely to report associated depression, Supplement Table 4a. Although confidence intervals were wide with probability values greater than 0.01, there was some evidence that the reported percentages of those with moderately severe (coefficient = 0.30 (95% CI = 0.07 to 0.53, *p* = 0.014) and severe (coefficient = 0.28 (95% CI = 0.08 to 0.48, *p =* 0.01) depression increased with length of time in confinement from zero to ten weeks, but the reported percentages of those with severe anxiety did not (coefficient = 0.195 (95% CI = -0.22 to 0.61).

### Increasing the risk of reported heavy drinking and substance use

Path analyses found that the same risk and protective factors for reported depression, anxiety and lack of mental well-being (as included in Tables 3 and 4) had similar associations with the likelihood of reporting heavy drinking and substance use, Supplement Table 6a, with odds ratios becoming closer to 1.0 when reported depression, anxiety and lack of mental well-being were respectively added to the models, Supplement Table 6b. Thus, much of the associations between the risk and protective factors and heavy drinking and substance use seemed to operate through the indirect paths of increased likelihood of depression, anxiety and lack of mental well-being, which, in turn, were associated with increased reporting of heavy drinking and substance use.

### Categorical principal components analyses

The categorical principal components analyses identified similar sets of risk and protective factors reported in Tables 3 and 4 associated with the likelihood of reporting depression, anxiety and lack of mental well-being, Supplement Figs. 1–3. Based on the created scales from the identified sets of factors, we found relationships with likelihoods of reported depression and anxiety. The greater the number of items of worry reported, the greater the likelihood of reporting depression (OR = 1.32, 95% CI = 1.31 to 1.34), including severe depression (OR = 1.48, 95% CI = 1.44 to 1.52), and anxiety (OR = 1.41, 95% CI = 1.40 to 1.43), including severe anxiety (OR = 1.45, 95% CI = 1.42 to 1.47). Conversely, the greater the number of protective factors reported, the less the likelihood of reporting depression (OR = 0.58, 95% CI = 0.57 to 0.60), including severe depression (OR = 0.46, 95% CI = 0.42 to 0.50), and anxiety (OR = 0.63, 95% CI = 0.61 to 0.65), including severe anxiety (OR = 0.63, 95% CI = 0.60 to 0.65).

## Discussion

We found that reported mental ill-health in the general Catalan population tripled compared with before confinement, with large increases in the use of prescribed and non-prescribed hypnosedatives but no increases in tobacco or alcohol use. Between six to ten weeks into confinement, our reported prevalences for depression (22.8%) and anxiety (26.9%) are slightly higher than those reported from one other study in Spain of 3480 people 2 weeks into confinement, in which prevalences were 18.7% for depression and 21.6% for anxiety [24]. Within the studied population, women and the younger population had a greater increased likelihood of reporting mental ill-health, and the older and retired population a smaller increased likelihood.

Our prevalence findings are similar to the results of the two meta-analyses that found, during confinement, reported prevalences of 25% for both depression [9] and anxiety [10]. Our prevalence estimates were derived from symptom-based scales (for depression, the Patient Health Questionnaire [16], and for anxiety, the Generalized Anxiety Disorder Scale [17]), similar to the scales most commonly used in the meta-analyses [9, 10]. Our findings of risk and protective factors are also very similar to those of the meta-analyses, where these were reported. As we found, the meta-analyses reported that those are greater risk of mental ill-health were women, younger adults, students, those with lower levels of education, perceived high risk of job loss, lack of social support, presence of existing health problems, and worry about oneself or family members getting COVID-19. The meta-analyses, as our own study, also found that health and other front line workers were not at increased risk of mental-ill-health. Similar to our own findings, the meta-analysis of prevalence of anxiety found that increased levels of alcohol consumption were associated with increased anxiety. Of the few protective factors considered in the meta-analyses, similar to our own findings, pursuing hobbies decreased the likelihood of reporting mental-ill health.

One of the unexpected findings of our survey was that the prevalence of reported mental ill-health was not affected by when the survey was completed during the 30-day sampling period, which covered weeks 6 through 10 of confinement. Since essential workers could continue work outside the home during confinement, respondents reported a range of tiem in confinement of between zero and ten weeks; the length of time in confinement within this range was not associated with the prevalence of reported mental ill-health, with the exception of moderately severe or severe depression which showed a small increase over reported length of time in confinement. These findings are in contrast with the other Spanish study which found that distress levels increased in parallel with the number of days without leaving the house [25].

The study has unpacked a range of conditions and coping behaviours that were associated with decreased likelihood of reporting mental ill-health, such as spending time on oneself, and following a routine; and factors associated with an increased likelihood of reporting mental ill-health, such as difficulties in all living together at home, or facing uncertainty. For all factors, the associations were more impactful the more severe the reported depression and anxiety. Further, the higher the number of reported risk factors, the greater was the likelihood of reporting depression and anxiety; and the higher the number of reported protective factors, the smaller was the likelihood of reporting depression and anxiety. The study provides an evidence base that can help build preventive programmes that mitigate mental ill-health in future similar situations.

### Strengths and limitations of the study

The data is a convenience sample, typical of modern web-based population studies that have replaced traditional random-digit telephone surveys because of declining response rates and problems of defining sampling frames [26]. A large sample size of 37,810 adults was achieved. The sample was overrepresented by women, middle-aged adults, and those with higher education, similar to the representativeness of other reported surveys during COVID-19 [3, 4, 24]. Dealing with non-probability sampling problems [27], our study: detailed the sampling strategy; applied statistical analyses and modelling to ensure interpretation and avoid sampling bias; used weighting procedures with the same population data to allow comparing the study results with those of previous surveys [13–15]; and, used standardised measures that are reliable and stable over time to be able to compare to pre-pandemic baseline data from the same population. Despite all of this, one cannot be certain that self-selection in answering the survey might have led to either an increased or a decreased number of responses from those most affected. We are reporting associations and cannot determine directions of causality. Whilst worry about a range of factors could be associated with an increased likelihood of reporting mental ill-health, it is possible that increased anxiety or depression could lead to increased worry. Nevertheless, as many associations are found in expected directions of causality, it is not inappropriate to recommend a range of actions that might mitigate mental ill-health during further timers of confinement.

### Preparing for future pandemics

Understanding of the factors associated with mental well-being and poorer mental health help pinpoint a set of policy measures to support preparedness (Table 5).

**Table 5:** Preparing for future waves

- Targeted approaches for vulnerable groups, including younger people	
- Match increased mental ill-health prevalences with service provision	
- Leverage e-health for intervention delivery	
- Support mental health of and via health professionals	
- Increase mental health literacy of the population	
- Promote resilience to help deal with uncertainty	
- Policy development to address structural measures	

#### Targeted approaches for younger people

Similar to the results of other studies, younger people reported a significantly higher prevalence of symptoms [3, 24, 28]. This might explain the recent disinhibition behaviours that have been observed once confinements have been lifted, resembling compensation or countering behaviours. This calls for the need to pay more attention to younger populations through programmes that: target their worries; ensure increased awareness of risk and protective factors and the mental health consequences of likely recurring pandemic waves and confinements; and, mainstream these through policies and other delivery mechanisms such as online resource packs.

#### Identification and services provision, including leveraging e-health

The three-fold increase in symptomatology highlights a two-sided problem: first, given the upsurge, the consequence is a likely lack of identification of cases and gaps in service provision for those in need. And, second, when mental health problems are addressed, there will be an inevitable increase in demand for mental health services. This is further evidenced when establishing a parallel with the epidemic of Severe Acute Respiratory Syndrome, in which beyond the increase of mental ill-health, confinement worsened pre-existing mental illness, and implied persistence of symptomatology following the pandemic [29]. It follows that as part of future preparedness efforts, it is essential to develop: mechanisms for ongoing surveillance of mental health; improved screening for mental disorders; efficient links between primary care, community, and hospital services maximising the use of technological advances; and, leveraging telemedicine and e-health innovations to deliver mental health care.

#### Mental health “of” and “via” health professionals

There are three urgent needs to support health professionals. First, other studies have found over the longer term significant increases in mental ill-health amongst health professionals, evidencing a need to increase awareness of the pandemic’s impact on health professionals’ own mental health, normalizing increased symptomatology and the development of future disorders such as post-traumatic stress disorder [4, 30], and providing assessment tools and anonymous or self-referral mechanisms for treatment. Second, there is a need to increase mental health literacy of health professionals, especially those in primary health care, to be aware of the expected increases of symptomatology and morbidity in their patients, and providing an easy care-pathway for identification and referral of patients with mental health needs [31, 32]. Finally, training modules for mental health providers should be offered (using different formats) to develop capacity and capabilities to leverage online or telemedicine formats for assessment and intervention provision, helping to move towards blended care models [33].

#### Mental health literacy in the general population

Mental health literacy needs to be increased in the general population through raising awareness of the consequences of the current pandemic on mental well-being, destigmatising mental illness, and preparing for forthcoming similar situations. As such, mental health literacy packs should include: information on the “normality” of increased depression and anxiety symptoms and reduced mental well-being in confinement situations; recommendations for reducing risk factors, such as limit time accessing COVID-19 related information; and, highlighting protective factors such as the importance of enhancing social support, dedicating time to oneself, developing activities with family. Equally important is the quality of information in media and social networks, and efforts should be addressed to these, given their effects on the population.

#### Resilience building for all

The lack of mental well-being in three quarters of the population calls for immediate programmes to increase resilience. Such programmes should build on existing positive psychology resilience building. Resilience programmes should also build on new evidence to address protective factors stemming from COVID-19 confinement research such as those identified in this study: the importance of, and how to follow a routine; the relevance of earmarking even a short time for oneself; how to control negative spiral thinking; and, making use of support networks. A study providing support to health professionals in China identified the demand and use of such approaches, showing 36% accessing psychological materials and 50% accessing psychological resources available through media/on-line [30]. Beyond health workers, it is important to provide already existing evidence-based mental health promotion and resilience interventions [23, 34], leveraging e-health innovations and platforms developed during the pandemic. Concurrently, comprehensive crisis prevention and intervention systems need to be developed to reduce psychological distress and to prevent further mental health problems. Research on the best delivery and implementation mechanisms, best types of approach and maximizing reach of interventions should be simultaneously studied [11].

#### Structural support to minimize risk factors

In preparedness plans for future pandemics, it is essential to address structural risk factors that precipitate poor mental health, such as how to support adults having to balance working from home with having to care for children. Better educational schemes and pre-set routines and sources for children’s structured learning are essential in face of future confinements. Similarly, economic and labour uncertainty and strain will remain strongly associated with continued poor mental health. Measures to support the disadvantaged, and to structurally minimize economic worries are urgently needed.

### Need for integrated policy approaches

When submitting this paper, new confinements are being put in place in different cities around the world, signalling we are in this for the long haul. This is why this pandemic should become the turning point and the opportunity for a long needed social transformation, including: a focus on promoting mental well-being through resilience; strengthening health systems and mental health policies; and, integrating responses in a more horizonal comprehensive set of community policies and programmes.

## Conclusions

The survey results indicated a clear negative impact of confinement on mental ill-health. Weighted prevalence of reported depression, anxiety and lack of mental well-being was, respectively, 23, 26, and 75%, each three-fold higher than before confinement. The use of prescribed hypnosedatives was two-fold and of non-prescribed hypnosedatives ten-fold higher than in 2018. Women, younger adults and students were considerably more likely, and older and retired people considerably less likely to report mental ill-health.

The survey also identified a range of associated factors associated with reported prevalence of mental-ill-health. High levels of social support, dedicating time to oneself, following a routine, and undertaking relaxing activities were associated with half the likelihood of reported mental ill-health. Worrying about problems living at home, the uncertainty of when normality would return, and job loss were associated with more than one and a half times the likelihood of mental ill-health.

Should future confinements be put in place, including increasing the mental health literacy of the population through extensive communication campaigns; promoting resilience to help deal with uncertainty through existing positive psychology resilience building programmes both for the general population and for health care professionals; and, address structural measures through policy development that deal with educational schemes for children and that deal with economic and labour uncertainty, in particular for disadvantaged groups.

## Supplementary Information

**Additional file 1.**

## Data Availability

The data set used and analysed during the current study is available from the first author (Eva Jané Llopis (eva.jane@esade.edu) on reasonable request.

## Abbreviations

AUDIT-C: Alcohol Use Disorders Identification Test, three alcohol consumption questions
CI: Confidence Interval
OR: Odds ratio
