# Supplementary material for: Mental ill-health during COVID-19 confinement
Source: BMC Psychiatry. 2021 Apr 14;21:194. doi: 10.1186/s12888-021-03191-5 (PMC8045571; doi:10.1186/s12888-021-03191-5)
Supplement: Supplementary file 1 — Additional file 1. [file 12888_2021_3191_MOESM1_ESM.docx]

**Supplement Table 1** Variables used in the analyses. Their definitions and distributions in the survey population

|  | **Present** | | **Absent** | |
| --- | --- | --- | --- | --- |
|  |  | |  |  |
| **Variables** | **Number** | **Percent** | **Number** | **Percent** |
| Worry about being infected by Covid-19 (onself or family member)^a^ | 18570 | 49.1 | 19281 | 50.9 |
| Worry about being alone and not being able to take care of oneself ^a^ | 4152 | 11.0 | 33699 | 89.0 |
| Worry about family members who are alone ^a^ | 12811 | 33.8 | 25040 | 66.2 |
| Worry about having a health emergency and not receiving any care (oneself or family member) ^a^ | 19008 | 50.2 | 18843 | 49.8 |
| Worry about job loss ^a^ | 8938 | 23.6 | 28913 | 76.4 |
| Worry about economic difficulties to buy food, pay rent, electricity etc. ^a^ | 7882 | 20.8 | 29969 | 79.2 |
| Worry about loss of an academic year or the educational future of own children ^a^ | 5745 | 15.2 | 32106 | 84.8 |
| Worry about inability to combine having to work from home while taking care of family (children, dependent persons, etc) ^a^ | 5195 | 13.7 | 32656 | 86.3 |
| Worry about children being anxious, children not knowing what to do, leading to tensions and bad behaviour ^a^ | 4850 | 12.8 | 33001 | 87.2 |
| Worry about not being able to go out of the house nor visit loved ones ^a^ | 12291 | 32.5 | 25560 | 67.5 |
| Worry about the uncertainty of when and how normality will return ^a^ | 14493 | 38.3 | 23358 | 61.7 |
| Worry about the impact the situation will have in the community (e.g., own neighborhood) ^a^ | 12770 | 33.7 | 25081 | 66.3 |
| Worry about problems living at home ^a^ | 2652 | 7.0 | 35199 | 93.0 |
| Worry about violence at home^a^ | 1288 | 3.4 | 36563 | 96.6 |
| Follow a routine^b^ | 23544 | 62.2 | 14307 | 37.8 |
| Talk with family or friends via phone or videoconference^b^ | 23860 | 63.0 | 13991 | 37.0 |
| Watch news about Covid-19 very often (television, social media, etc)^b^ | 20983 | 55.4 | 16868 | 44.6 |
| Take the opportunity to do housework (e.g., DIY projects, cooking, etc)^b^ | 12643 | 33.4 | 25208 | 66.6 |
| Spend time outdoors (patio, balcony) or looking outside (street, sky, etc.)^b^ | 19931 | 52.7 | 17920 | 47.3 |
| Undertake relaxing activities (e.g., listen to music)^b^ | 14203 | 37.5 | 23648 | 62.5 |
| Dedicate time to oneself^b^ | 6239 | 16.5 | 31612 | 83.5 |
| Spend more time doing activities with family^b^ | 12226 | 32.3 | 25625 | 67.7 |
| Take the opportunity to learn new things (new studies, hobbies, etc) ^b^ | 3768 | 10.0 | 34083 | 90.0 |
| Take the opportunity to advance paperwork or other delayed activities ^b^ | 6203 | 16.4 | 31648 | 83.6 |
| Does not eat more to cope with situation^c^ | 14045 | 37.1 | 23806 | 62.9 |
| Does not drink more alcohol to cope with the situation^c^ | 28217 | 74.5 | 9634 | 25.5 |
| Has a job that increases risk of COVID-19^d^ | 7930 | 21.0 | 29921 | 79.0 |
| Suffer from chronic diseases that increase own risk of COVID-19 infection^d^ | 5279 | 13.9 | 32572 | 86.1 |
| Have had COVID-19 with mild symptomatology ^d^ | 3484 | 9.2 | 34367 | 90.8 |
| Have had COVID-19 with severe symptomatology ^d^ | 330 | 0.9 | 37521 | 99.1 |
| Have family or friends with risk factors for COVID-19 (e.g., older age, previous diseases) ^d^ | 19299 | 51.0 | 18552 | 49.0 |
| Have family or friends with COVID-19 but not hospitalised ^d^ | 10591 | 28.0 | 27260 | 72.0 |
| Have family or friends with COVID-19 hospitalised ^d^ | 8230 | 21.7 | 29621 | 78.3 |
| Have family or a close friend who has died because of COVID-19 ^d^ | 6766 | 17.9 | 31085 | 82.1 |
| Have children and they take up half of my time or less^d^ | 6970 | 18.4 | 30881 | 81.6 |
| Have children and they take up most of my time^d^ | 5934 | 15.7 | 31917 | 84.3 |
| Be responsible for a dependent person/s and they take up half of my time or less^d^ | 1817 | 4.8 | 36034 | 95.2 |
| Be responsible for a dependent person/s and they take up most of the time^d^ | 483 | 1.3 | 37368 | 98.7 |
| High social support v low social support (3-point scale) | 12240 | 61.9 | 7526 | 38.1 |
| Moderate social support v low social support (3-point scale) | 18085 | 70.6 | 7526 | 29.4 |
| Spending more than 2 hours a day reading COVID-19 news or information^d^ | 7135 | 18.9 | 30716 | 81.1 |
| Having a balcony, terrace, or garden^d^ | 34463 | 91.0 | 3388 | 9.0 |
| Weeks in confinement (9+ versus <9) | 1578 | 4.2 | 36273 | 95.8 |
| Number of people in house (5+ versus <5) | 3775 | 10.0 | 34076 | 90.0 |
| Number of rooms in house (4+ versus <4) | 18306 | 48.4 | 19545 | 51.6 |
| Being a woman versus not | 28034 | 74.1 | 9817 | 25.9 |
| Primary and secondary education versus postgraduate education (3-point scale) | 16159 | 67.1 | 7923 | 32.9 |
| University education versus postgraduate education (3-point scale) | 13769 | 63.5 | 7923 | 36.5 |
| Aged 65+ versus aged 15-44 years (3-point scale) | 4517 | 23.8 | 14457 | 76.2 |
| Aged 45-64 versus aged 15-44 years (3-point scale) | 18877 | 56.6 | 14457 | 43.4 |
| Own job future is very likely to get worse^e^ | 6463 | 17.1 | 31388 | 82.9 |
| Worried about economic consequences of COVID-19^f^ | 8934 | 23.6 | 28917 | 76.4 |
| Being a health worker versus not | 4503 | 11.9 | 33348 | 88.1 |
| Being another front-line worker (excluding health worker) versus not | 4393 | 11.6 | 33458 | 88.4 |
| Being another worker (excluding health worker and other frontline worker) versus not | 14147 | 37.4 | 23704 | 62.6 |
| Being on sick leave versus not | 1340 | 3.5 | 36511 | 96.5 |
| Being unemployed versus not | 5129 | 13.6 | 32722 | 86.4 |
| Being a student versus not | 1107 | 2.9 | 36744 | 97.1 |
| Being retired versus not | 5127 | 13.5 | 32724 | 86.5 |

^a^Worry a lot (score 4 on a 4-point Likert scale from 1, not worried to 4, worried a lot) versus not score 4

^b^Almost every day (score 4 on a 4-point Likert scale from 1, not any day to 4, almost every day) versus not score 4

^c^Not any day (score 1 on a 4-point Likert scale from 1, not any day to 4, almost every day) versus not score 1

^d^Present versus absent

^e^Own job future very likely to get worse (score 5 on a 5-point Likert scale from 1, very likely to get better to 5, very likely to get worse) versus not score 5

^f^Very worried (score 10 on a 10-point Likert scale from 1, not at all worried to 10, very worried) versus not score 10

**Supplement Table 2** Demographic characteristics of Catalan and survey populations.

|  |  | **Catalan Population** | **Survey population** | |
| --- | --- | --- | --- | --- |
|  |  | % | % | n |
| **Gender** | Female | 51.5 | 74.1 | 28,034 |
|  | Male | 48.5 | 25.6 | 9,685 |
|  | Other | Not reported | 0.3 | 132 |
| **Age** | 15-44 years | 44.8 | 38.0 | 14,457 |
|  | 45-64 years | 32.9 | 50.1 | 18,877 |
|  | 65+ years | 21.3 | 11.8 | 4,517 |
| **Achieved educational level** | Primary | 19.4 | 5.8 | 2,200 |
|  | Secondary | 57.6 | 36.9 | 13,959 |
|  | University | 22.9 | 36.4 | 13,769 |
|  | Postgraduate |  | 20.9 | 7,923 |
| **Employment category** | Employed | 54.8 | 60.9 | 23,043 |
|  | Unempoyed | 6.8 | 13.6 | 5,129 |
|  | Retired | 21.6 | 13.5 | 5,127 |
|  | Disabled | 2.0 | 3.5 | 1,340 |
|  | Student | 6.1 | 2.9 | 1,107 |
|  | Other | 8.7 | 5.6 | 2,105 |

**Supplement Table 3a** Reported present mental health outcomes by socio-demographic variables

Each socio-demographic dummy variable coded as present/absent

N = sample size

Proportion% = proportion (%) of socio-demographic dummy variable with reported mental health outcome; 95% confidence intervals from bootstrapping, n=1000.

OR = odds ratio (95% CI) for reported mental health outcome, with socio-demographic dummy variable present versus absent; P for OR, the probability value for the odds ratio.

| **Socio-demographic group** | **Dummy variable** | **Depression** | | | | **Anxiety** | | | | **Lack of mental well-being** | | | |
| --- | --- | --- | --- | --- | --- | --- | --- | --- | --- | --- | --- | --- | --- |
|  |  | **N** | **Proportion (%)** | **OR (95%CI)** | **P for OR** | **N** | **Proportion (%)** | **OR (95%CI)** | **P for OR** | **N** | **Proportion (%)** | **OR (95%CI)** | **P for OR** |
| **Gender** | **Female** | 23410 | 26.56  (26.02 to 27.12) | 1.71  (1.60 to 1.83) | 0.0000 | 23721 | 30.26  (29.63 to 30.83) | 1.75  (1.64 to 1.86) | 0.0000 | 27818 | 80.94  (80.49 to 81.38) | 1.65  (1.56 to 1.74) | 0.0000 |
|  | **Male** | 8325 | 15.93  (15.17 to 16.69) | 0.57  (0.53 to 0.61) | 0.0000 | 8327 | 18.65  (17.78 to 19.47) | 0.56  (0.53 to 0.60) | 0.0000 | 9618 | 69.52  (68.55 to 70.44) | 0.60  (0.57 to 0.64) | 0.0000 |
|  | **Other** | 114 | 41.74  (32.26 to 50.98) | 1.98  (1.35 to 2.91) | 0.0004 | 112 | 40.71  (31.37 to 49.44) | 1.59  (1.08 to 2.34) | 0.0182 | 130 | 83.97  (77.61 to 90.08) | 1.34  (0.82 to 2.17) | 0.2405 |
| **Age (years)** | **15-44** | 11992 | 32.27  (31.40 to 33.13) | 2.07  (1.97 to 2.19) | 0.0000 | 12343 | 35.32  (34.48 to 36.20) | 1.92  (1.82 to 2.02) | 0.0000 | 14350 | 86.06  (85.50 to 86.64) | 2.16  (2.05 to 2.29) | 0.0000 |
|  | **45-64** | 16103 | 20.57  (19.96 to 21.18) | 0.68  (0.64 to 0.72) | 0.0000 | 16213 | 23.84  (23.17 to 24.48) | 0.69  (0.65 to 0.72) | 0.0000 | 18732 | 75.59  (75.02 to 76.21) | 0.76  (0.72 to 0.80) | 0.0000 |
|  | **65+** | 3754 | 10.87  (9.92 to 11.85) | 0.38  (0.34 to 0.42) | 0.0000 | 3604 | 15.32  (14.09 to 16.48) | 0.48  (0.43 to 0.52) | 0.0000 | 4484 | 62.42  (60.90 to 63.81) | 0.45  (0.43 to 0.49) | 0.0000 |
| **Highest education level** | **Primary** | 1751 | 27.74  (25.68 to 29.81) | 1.57  (1.41 to 1.76) | 0.0000 | 1708 | 35.10  (32.84 to 37.44) | 1.83  (1.65 to 2.03) | 0.0000 | 2167 | 70.60  (68.76 to 72.54) | 0.80  (0.72 to 0.88) | 0.0000 |
|  | **Secondary** | 11372 | 28.02  (27.19 to 28.86) | 1.48  (1.40 to 1.56) | 0.0000 | 11565 | 31.44  (30.63 to 32.27) | 1.43  (1.36 to 1.51) | 0.0000 | 13867 | 77.68  (76.99 to 78.36) | 0.98  (0.93 to 1.04) | 0.5308 |
|  | **University** | 11736 | 20.76  (19.99 to 21.48) | 0.77  (0.73 to 0.82) | 0.0000 | 11805 | 24.10  (23.35 to 24.90) | 0.78  (0.74 to 0.82) | 0.0000 | 13669 | 78.34  (77.66 to 78.97) | 1.06  (1.01 to 1.12) | 0.0204 |
|  | **Postgraduate** | 6990 | 21.23  (20.24 to 22.20) | 0.71  (0.67 to 0.76) | 0.0000 | 7082 | 23.93  (22.94 to 24.93) | 0.70  (0.66 to 0.75) | 0.0000 | 7863 | 80.15  (79.31 to 81.00) | 1.02  (0.96 to 1.09) | 0.4629 |
| **Occupation group** | **Health worker** | 3942 | 24.91  (23.53 to 26.32) | 1.05  (0.97 to 1.14) | 0.2564 | 4020 | 31.29  (29.77 to 32.74) | 1.27  (1.18 to 1.37) | 0.0000 | 4466 | 80.99  (79.80 to 82.18) | 1.02  (0.94 to 1.11) | 0.6363 |
|  | **Other worker** | 3694 | 20.60  (19.32 to 21.86) | 0.73  (0.68 to 0.77) | 0.0000 | 3725 | 26.50  (25.09 to 27.86) | 0.79  (0.75 to 0.84) | 0.0000 | 4362 | 75.17  (73.85 to 76.39) | 1.04  (0.99 to 1.10) | 0.1485 |
|  | **Other front-line worker** | 12077 | 20.87  (20.18 to 21.61) | 0.74  (0.68 to 0.81) | 0.0000 | 12257 | 24.69  (23.95 to 25.40) | 0.90  (0.83 to 0.98) | 0.0127 | 14037 | 80.12  (79.47 to 80.78) | 0.77  (0.71 to 0.83) | 0.0000 |
|  | **On sick leave** | 1074 | 42.09  (39.11 to 45.24) | 2.35  (2.07 to 2.67) | 0.0000 | 1118 | 38.55  (35.69 to 41.46) | 1.65  (1.46 to 1.87) | 0.0000 | 1329 | 83.52  (81.61 to 85.56) | 1.46  (1.26 to 1.69) | 0.0000 |
|  | **Unemployed** | 4194 | 33.73  (32.22 to 35.15) | 1.35  (1.25 to 1.45) | 0.0000 | 4342 | 35.44  (34.08 to 36.73) | 1.20  (1.12 to 1.29) | 0.0000 | 5111 | 81.69  (80.58 to 82.70) | 1.09  (1.00 to 1.18) | 0.0416 |
|  | **Student** | 863 | 54.72  (51.40 to 58.10) | 2.35  (2.04 to 2.72) | 0.0000 | 872 | 44.82  (41.54 to 48.10) | 1.27  (1.10 to 1.47) | 0.0010 | 1081 | 91.47  (89.87 to 93.09) | 1.92  (1.54 to 2.39) | 0.0000 |
|  | **Other** | 1747 | 28.07  (26.00 to 30.06) | 1.10  (0.98 to 1.22) | 0.1057 | 1733 | 30.55  (28.28 to 32.75) | 1.03  (0.92 to 1.15) | 0.5960 | 2091 | 78.55  (76.85 to 80.44) | 0.96  (0.86 to 1.07) | 0.4453 |
|  | **Retired** | 4258 | 11.62  (10.68 to 12.55) | 0.72  (0.61 to 0.84) | 0.0000 | 4093 | 14.97  (13.84 to 15.99) | 0.63  (0.54 to 0.72) | 0.0000 | 5089 | 63.83  (62.44 to 65.15) | 0.79  (0.71 to 0.88) | 0.0000 |
| **TOTAL** | **Unweighted** | 31849 | 23.84  (23.33 to 24.31) | |  | 32160 | 27.29  (26.84 to 27.73) | |  | 37566 | 78.02  (77.60 to 78.44) | |  |
|  | **Weighted^a^** |  | 22.82  (22.11 to 23.55) | |  |  | 26.88  (26.06 to 27.69) | |  |  | 74.76  (73.65 to 75.97) | |  |
| **Regression, Durbin Watson^b^** |  |  | **Coeff. (95%CI)** | **Durbin Watson** |  |  | **Coeff. (95%CI)** | **Durbin Watson** |  |  | **Coeff. (95%CI)** | **Durbin Watson** |  |
|  |  | | 0.033  (-0.203 to 0.269) | 2.1 |  |  | 0.148  (-0.100 to 0.396) | 1.775 |  |  | 0.052  (-0.12 to 0.222) | 2.009 |  |

^a^Weighted for sex, age and educational level of Catalan population

^b^Linear regression of dependent variable with day over all days of data collection; Durbin Watson test for auto-correlation

**Supplement Table 3b** Reported AUDIT-C positive and substance use during previous 30 days, and, for smokers, smoked more during confinement, by socio-demographic variables

Each socio-demographic dummy variable coded as present/absent

N = sample size

Proportion% = proportion (%) of socio-demographic dummy variable with reported mental health outcome; 95% confidence intervals from bootstrapping, n=1000.

OR = odds ratio (95% CI) for reported mental health outcome, with socio-demographic dummy variable present versus absent; P for OR, the probability value for the odds ratio.

| **Socio-demographic group** | **Dummy variable** | **AUDIT-C positive (5+)** | | | | **Smokes** | | | | **Smoked more cigarettes during confinement** | | | | **Cannabis** | | | |
| --- | --- | --- | --- | --- | --- | --- | --- | --- | --- | --- | --- | --- | --- | --- | --- | --- | --- |
|  |  | **N** | **Proportion (%)** | **OR (95%CI)** | **P for OR** | **N** | **Proportion (%)** | **OR (95%CI)** | **P for OR** | **N** | **Proportion (%)** | **OR (95%CI)** | **P for OR** | **N** | **Proportion (%)** | **OR (95%CI)** | **P for OR** |
| **Gender** | **Female** | 27569 | 5.22  (4.95 to 5.47) | 0.38  (0.35 to 0.41) | 0.0000 | 28015 | 22.37  (21.93 to 22.87) | 1.11  (1.05 to 1.18) | 0.0003 | 6991 | 45.28  (44.12 to 46.48) | 1.47  (1.33 to 1.63) | 0.0000 | 28015 | 2.32  (2.14 to 2.50) | 0.44  (0.38 to 0.49) | 0.0000 |
|  | **Male** | 9538 | 12.60  (11.9 to 13.2) | 2.64  (2.43 to 2.86) | 0.0000 | 9674 | 19.59  (18.82 to 20.39) | 0.90  (0.85 to 0.95) | 0.0003 | 2192 | 34.53  (32.46 to 36.63) | 0.67  (0.61 to 0.75) | 0.0000 | 9674 | 4.21  (3.83 to 4.63) | 2.19  (1.93 to 2.49) | 0.0000 |
|  | **Other** | 125 | 11.11  (5.88 to 16.67) | 1.67  (0.95 to 2.91) | 0.0726 | 131 | 24.24  (17.04 to 31.85) | 1.05  (0.70 to 1.57) | 0.8177 | 40 | 45.00  (28.95 to 62.16) | 1.04  (0.55 to 1.95) | 0.9085 | 131 | 13.64  (7.76 to 20.14) | 4.36  (2.61 to 7.28) | 0.0000 |
| **Age (years)** | **15-44** | 14219 | 6.62  (6.19 to 7.03) | 0.97  (0.89 to 1.06) | 0.5095 | 14426 | 24.50  (23.83 to 25.20) | 1.36  (1.30 to 1.44) | 0.0000 | 4024 | 47.89  (46.34 to 49.47) | 1.45  (1.33 to 1.57) | 0.0000 | 14426 | 5.23  (4.86 to 5.62) | 4.45  (3.89 to 5.09) | 0.0000 |
|  | **45-64** | 18582 | 7.58  (7.22 to 7.97) | 1.14  (1.05 to 1.23) | 0.0017 | 18877 | 22.07  (21.47 to 22.64) | 1.02  (0.97 to 1.07) | 0.4886 | 4631 | 40.79  (39.23 to 42.23) | 0.84  (0.77 to 0.91) | 0.0000 | 18877 | 1.53  (1.36 to 1.71) | 0.35  (0.31 to 0.40) | 0.0000 |
|  | **65+** | 4431 | 6.88  (6.12 to 7.64) | 0.79  (0.69 to 0.89) | 0.0002 | 4517 | 11.03  (10.11 to 11.94) | 0.40  (0.36 to 0.44) | 0.0000 | 568 | 22.01  (18.63 to 25.44) | 0.39  (0.32 to 0.48) | 0.0000 | 4517 | 0.71  (0.47 to 0.97) | 0.19  (0.13 to 0.27) | 0.0000 |
| **Highest education level** | **Primary** | 2143 | 7.64  (6.43 to 8.69) | 1.05  (0.89 to 1.24) | 0.5475 | 2182 | 26.95  (25.06 to 28.89) | 1.55  (1.40 to 1.71) | 0.0000 | 633 | 47.39  (43.27 to 51.19) | 1.32  (1.12 to 1.56) | 0.0010 | 2182 | 2.41  (1.78 to 3.05) | 1.22  (0.92 to 1.63) | 0.1676 |
|  | **Secondary** | 13735 | 7.50  (7.04 to 7.92) | 1.06  (0.97 to 1.15) | 0.1821 | 13951 | 26.83  (26.02 to 27.56) | 1.61  (1.53 to 1.69) | 0.0000 | 4094 | 43.70  (42.08 to 45.27) | 1.06  (0.98 to 1.16) | 0.1580 | 13951 | 3.55  (3.23 to 3.87) | 1.46  (1.29 to 1.65) | 0.0000 |
|  | **University** | 13561 | 6.73  (6.29 to 7.16) | 0.94  (0.86 to 1.02) | 0.1512 | 13765 | 18.87  (18.24 to 19.53) | 0.78  (0.74 to 0.82) | 0.0000 | 2938 | 41.59  (39.71 to 43.45) | 0.96  (0.87 to 1.05) | 0.3340 | 13765 | 2.40  (2.14 to 2.65) | 0.85  (0.74 to 0.97) | 0.0150 |
|  | **Postgraduate** | 7793 | 7.04  (6.47 to 7.62) | 0.99  (0.89 to 1.09) | 0.8030 | 7922 | 15.97  (15.19 to 16.83) | 0.58  (0.54 to 0.62) | 0.0000 | 1558 | 40.41  (38.10 to 42.85) | 0.85  (0.76 to 0.95) | 0.0038 | 7922 | 2.49  (2.15 to 2.87) | 0.68  (0.58 to 0.80) | 0.0000 |
| **Occupation group** | **Health worker** | 4430 | 5.35  (4.67 to 6.05) | 0.78  (0.68 to 0.90) | 0.0007 | 4503 | 20.30  (19.03 to 21.45) | 0.98  (0.90 to 1.06) | 0.5739 | 1065 | 41.13  (38.21 to 43.86) | 0.89  (0.78 to 1.01) | 0.0760 | 4503 | 1.82  (1.45 to 2.23) | 0.65  (0.51 to 0.82) | 0.0003 |
|  | **Other worker** | 4325 | 8.44  (7.56 to 9.27) | 1.08  (0.99 to 1.18) | 0.0677 | 4393 | 25.95  (24.77 to 27.26) | 0.90  (0.85 to 0.95) | 0.0001 | 1262 | 43.11  (40.38 to 45.97) | 1.10  (1.00 to 1.20) | 0.0416 | 4393 | 3.03  (2.52 to 3.56) | 0.87  (0.76 to 1.00) | 0.0449 |
|  | **Other front-line worker** | 13944 | 7.51  (7.07 to 7.99) | 1.10  (0.98 to 1.24) | 0.1078 | 14142 | 20.66  (20.00 to 21.30) | 1.17  (1.08 to 1.26) | 0.0000 | 3339 | 44.61  (42.92 to 46.44) | 0.99  (0.88 to 1.12) | 0.8800 | 14142 | 2.70  (2.45 to 2.99) | 0.90  (0.74 to 1.08) | 0.2634 |
|  | **On sick leave** | 1315 | 5.93  (4.64 to 7.23) | 0.83  (0.65 to 1.05) | 0.1137 | 1340 | 26.57  (24.21 to 29.13) | 1.16  (1.02 to 1.31) | 0.0218 | 381 | 48.82  (43.35 to 53.52) | 1.28  (1.04 to 1.58) | 0.0193 | 1340 | 3.28  (2.39 to 4.31) | 1.36  (0.99 to 1.85) | 0.0565 |
|  | **Unemployed** | 5063 | 8.18  (7.42 to 8.95) | 1.20  (1.07 to 1.35) | 0.0017 | 5128 | 31.20  (29.92 to 32.55) | 1.39  (1.30 to 1.49) | 0.0000 | 1757 | 46.50  (44.33 to 48.75) | 1.06  (0.95 to 1.18) | 0.2860 | 5128 | 5.67  (5.06 to 6.32) | 1.82  (1.57 to 2.10) | 0.0000 |
|  | **Student** | 1060 | 2.78  (1.82 to 3.77) | 0.36  (0.25 to 0.52) | 0.0000 | 1085 | 14.18  (12.06 to 16.38) | 0.37  (0.31 to 0.44) | 0.0000 | 200 | 28.50  (22.28 to 34.57) | 0.38  (0.28 to 0.52) | 0.0000 | 1085 | 4.43  (3.20 to 5.61) | 0.65  (0.48 to 0.88) | 0.0053 |
|  | **Other** | 2070 | 6.08  (4.96 to 7.15) | 0.90  (0.75 to 1.09) | 0.2747 | 2103 | 24.18  (22.34 to 26.15) | 1.00  (0.90 to 1.11) | 0.9989 | 546 | 44.14  (39.74 to 48.13) | 1.01  (0.85 to 1.20) | 0.9147 | 2103 | 2.71  (2.07 to 3.45) | 1.01  (0.77 to 1.33) | 0.9315 |
|  | **Retired** | 5025 | 7.14  (6.40 to 7.88) | 1.02  (0.84 to 1.23) | 0.8677 | 5126 | 11.74  (10.80 to 12.67) | 0.65  (0.57 to 0.75) | 0.0000 | 673 | 24.96  (21.71 to 28.53) | 0.75  (0.58 to 0.98) | 0.0327 | 5126 | 0.72  (0.50 to 0.97) | 0.57  (0.34 to 0.94) | 0.0279 |
| **TOTAL** | **Unweighted** | 37232 | 7.13  (6.87 to 7.42) | |  | 37820 | 21.67  (21.29 to 22.11) | |  | 9223 | 42.73  (41.80 to 43.76) | |  | 37820 | 2.84  (2.67 to 3.01) | |  |
|  | **Weighted^a^** |  | 9.05  (8.54 to 9.60) | |  |  | 22.99  (22.25 to 23.67) | |  |  |  |  |  |  | 3.65  (3.36 to 3.96) | |  |
| **Regression, Durbin Watson^a^** |  |  | **Coeff. (95%CI)** | **Durbin Watson** |  |  | **Coeff. (95%CI)** | **Durbin Watson** |  |  | **Coeff. (95%CI)** | **Durbin Watson** |  |  | **Coeff. (95%CI)** | **Durbin Watson** |  |
|  |  |  | -0.039  (-0.12 to 0.04) | 2.398 |  |  | 0.015  (-0.15 to 0.19) | 1.538 |  |  | -0.068  (-0.488 to 0.352) | 1.871 |  |  | 0.054  (-0.02 to 0.125) | 1.722 |  |

^a^Weighted for sex, age and educational level of Catalan population

^b^Linear regression of dependent variable with day over all days of data collection; Durbin Watson test for auto-correlation

**Supplement Table 3c** Reported use of non-prescription and prescription hypnosedatives during past 30 days by socio-demographic variables

Each socio-demographic dummy variable coded as present/absent

N = sample size

Proportion% = proportion (%) of socio-demographic dummy variable with reported mental health outcome; 95% confidence intervals from bootstrapping, n=1000.

OR = odds ratio (95% CI) for reported mental health outcome, with socio-demographic dummy variable present versus absent; P for OR, the probability value for the odds ratio.

| **Socio-demographic group** | **Dummy variable** | **Non-prescription hypnosedatives** | | | | **Prescription hypnosedatives** | | | |
| --- | --- | --- | --- | --- | --- | --- | --- | --- | --- |
|  |  | **N** | **Proportion (%)** | **OR (95%CI)** | **P for OR** | **N** | **Proportion (%)** | **OR (95%CI)** | **P for OR** |
| **Gender** | **Female** | 28015 | 8.08  (7.75 to 8.39) | 1.50  (1.36 to 1.66) | 0.0000 | 28015 | 18.93  (18.46 to 19.35) | 1.90  (1.77 to 2.04) | 0.0000 |
|  | **Male** | 9674 | 5.08  (4.65 to 5.51) | 0.64  (0.58 to 0.71) | 0.0000 | 9674 | 12.03  (11.32 to 12.69) | 0.52  (0.48 to 0.55) | 0.0000 |
|  | **Other** | 131 | 17.42  (11.20 to 24.39) | 2.55  (1.62 to 4.01) | 0.0001 | 131 | 23.48  (16.51 to 30.97) | 1.68  (1.11 to 2.53) | 0.0132 |
| **Age (years)** | **15-44** | 14426 | 8.50  (8.07 to 8.97) | 1.25  (1.15 to 1.35) | 0.0000 | 14426 | 11.41  (10.90 to 11.89) | 0.48  (0.45 to 0.51) | 0.0000 |
|  | **45-64** | 18877 | 7.21  (6.83 to 7.57) | 0.97  (0.90 to 1.05) | 0.4498 | 18877 | 20.01  (19.44 to 20.55) | 1.47  (1.39 to 1.55) | 0.0000 |
|  | **65+** | 4517 | 4.25  (3.69 to 4.87) | 0.57  (0.49 to 0.67) | 0.0000 | 4517 | 23.84  (22.62 to 25.08) | 1.74  (1.61 to 1.88) | 0.0000 |
| **Highest education level** | **Primary** | 2182 | 7.45  (6.34 to 8.58) | 1.13  (0.95 to 1.33) | 0.1651 | 2182 | 24.14  (22.33 to 25.78) | 1.38  (1.24 to 1.53) | 0.0000 |
|  | **Secondary** | 13951 | 7.36  (6.92 to 7.79) | 1.02  (0.94 to 1.10) | 0.7060 | 13951 | 19.53  (18.85 to 20.12) | 1.31  (1.24 to 1.38) | 0.0000 |
|  | **University** | 13765 | 7.01  (6.59 to 7.44) | 0.93  (0.86 to 1.01) | 0.0826 | 13765 | 16.01  (15.42 to 16.65) | 0.83  (0.78 to 0.88) | 0.0000 |
|  | **Postgraduate** | 7922 | 7.86  (7.28 to 8.46) | 1.04  (0.95 to 1.14) | 0.4034 | 7922 | 13.13  (12.38 to 13.87) | 0.76  (0.70 to 0.81) | 0.0000 |
| **Occupation group** | **Health worker** | 4503 | 9.75  (8.88 to 10.67) | 1.32  (1.18 to 1.48) | 0.0000 | 4503 | 17.41  (16.28 to 18.49) | 1.15  (1.06 to 1.26) | 0.0012 |
|  | **Other worker** | 4393 | 6.85  (6.14 to 7.59) | 0.85  (0.78 to 0.93) | 0.0002 | 4393 | 14.05  (13.01 to 15.04) | 0.68  (0.64 to 0.72) | 0.0000 |
|  | **Other front-line worker** | 14142 | 7.01  (6.58 to 7.42) | 0.90  (0.79 to 1.02) | 0.0868 | 14142 | 13.05  (12.46 to 13.59) | 0.82  (0.75 to 0.90) | 0.0000 |
|  | **On sick leave** | 1340 | 9.25  (7.66 to 10.79) | 1.26  (1.04 to 1.52) | 0.0179 | 1340 | 44.25  (41.65 to 46.88) | 3.84  (3.43 to 4.31) | 0.0000 |
|  | **Unemployed** | 5128 | 8.56  (7.79 to 9.29) | 1.11  (1.00 to 1.24) | 0.0570 | 5128 | 17.14  (16.12 to 18.15) | 1.03  (0.95 to 1.12) | 0.4356 |
|  | **Student** | 1085 | 7.41  (5.92 to 8.90) | 0.83  (0.66 to 1.06) | 0.1369 | 1085 | 6.32  (4.92 to 7.76) | 0.42  (0.33 to 0.54) | 0.0000 |
|  | **Other** | 2103 | 8.27  (7.16 to 9.54) | 1.08  (0.92 to 1.26) | 0.3748 | 2103 | 23.71  (21.78 to 25.56) | 1.39  (1.25 to 1.54) | 0.0000 |
|  | **Retired** | 5126 | 4.49  (3.97 to 5.04) | 0.79  (0.64 to 0.99) | 0.0419 | 5126 | 23.68  (22.50 to 24.80) | 1.17  (1.03 to 1.32) | 0.0150 |
| **TOTAL** | **Unweighted** | 37820 | 7.34  (7.08 to 7.61) | |  | 37820 | 17.18  (16.79 to 17.57) | |  |
|  | **Weighted^a^** |  | 6.51  (6.13 to 6.86) | |  |  | 17.67  (17.05 to 18.37) | |  |
| **Regression, Durbin Watson^a^** |  |  | **Coeff. (95%CI)** | **Durbin Watson** |  |  | **Coeff. (95%CI)** | **Durbin Watson** |  |
|  |  |  | 0.014  (-0.052 to 0.080) | 2.102 |  |  | 0.059  (-0.11 to 0.231) | 1.728 |  |

^a^Weighted for sex, age and educational level of Catalan population

^b^Linear regression of dependent variable with day over all days of data collection; Durbin Watson test for auto-correlation

**Supplement Table 4a** Odds ratios (95% confidence intervals) and probability (p) values for associations between risk and protective variables and reported depression

|  | **TOTAL** | | **FOR WOMEN and MEN** | | | | **FOR THREE AGE GROUPS** | | | | | |
| --- | --- | --- | --- | --- | --- | --- | --- | --- | --- | --- | --- | --- |
|  |  | | **Women** | | **Men** | | **15-44** | | **45-64** | | **65+** | |
| **Variables** | **OR (95% CI)** | **P value** | **OR (95% CI)** | **P value** | **OR (95% CI)** | **P value** | **OR (95% CI)** | **P value** | **OR (95% CI)** | **P value** | **OR (95% CI)** | **P value** |
| Worry about being infected by Covid-19 (onself or family member)^a^ | 1.17  (1.10 to 1.24) | 0.000 | 1.17  (1.09 to 1.25) | 0.000 | 1.21  (1.04 to 1.40) | 0.011 | 1.12  (1.03 to 1.23) | 0.010 | 1.20  (1.09 to 1.31) | 0.000 | 1.41  (1.07 to 1.84) | 0.014 |
| Worry about being alone and not being able to take care of oneself ^a^ | 1.17  (1.07 to 1.28) | 0.000 | 1.21  (1.10 to 1.33) | 0.000 | 1.01  (0.81 to 1.25) | 0.937 | 1.16  (1.01 to 1.33) | 0.041 | 1.16  (1.03 to 1.32) | 0.016 | 1.25  (0.94 to 1.68) | 0.128 |
| Worry about family members who are alone ^a^ | 1.17  (1.10 to 1.25) | 0.000 | 1.18  (1.10 to 1.26) | 0.000 | 1.17  (1.01 to 1.36) | 0.038 | 1.15  (1.05 to 1.26) | 0.002 | 1.20  (1.09 to 1.31) | 0.000 | 1.13  (0.85 to 1.48) | 0.399 |
| Worry about having a health emergency and not receiving any care (oneself or family member) ^a^ | 1.09  (1.02 to 1.16) | 0.007 | 1.09  (1.01 to 1.17) | 0.022 | 1.13  (0.97 to 1.30) | 0.112 | 1.07  (0.98 to 1.17) | 0.122 | 1.12  (1.02 to 1.24) | 0.016 | 1.02  (0.78 to 1.34) | 0.864 |
| Worry about job loss ^a^ | 0.96  (0.89 to 1.04) | 0.300 | 0.97  (0.89 to 1.06) | 0.523 | 0.91  (0.76 to 1.09) | 0.301 | 0.98  (0.88 to 1.09) | 0.725 | 0.94  (0.84 to 1.06) | 0.332 | 0.93  (0.58 to 1.50) | 0.764 |
| Worry about economic difficulties to buy food, pay rent, electricity etc. ^a^ | 1.13  (1.05 to 1.23) | 0.002 | 1.15  (1.06 to 1.26) | 0.002 | 1.03  (0.86 to 1.24) | 0.715 | 1.19  (1.06 to 1.33) | 0.002 | 1.12  (0.99 to 1.26) | 0.063 | 0.79  (0.54 to 1.14) | 0.210 |
| Worry about loss of an academic year or the educational future of own children ^a^ | 0.96  (0.89 to 1.04) | 0.362 | 0.98  (0.90 to 1.07) | 0.655 | 0.92  (0.76 to 1.11) | 0.405 | 0.97  (0.86 to 1.09) | 0.612 | 0.94  (0.84 to 1.05) | 0.269 | 0.95  (0.62 to 1.45) | 0.803 |
| Worry about inability to combine having to work from home while taking care of family (children, dependent persons, etc) ^a^ | 1.10  (1.01 to 1.19) | 0.035 | 1.11  (1.01 to 1.22) | 0.026 | 1.00  (0.80 to 1.24) | 0.989 | 1.04  (0.92 to 1.17) | 0.539 | 1.24  (1.09 to 1.41) | 0.001 | 0.94  (0.53 to 1.64) | 0.818 |
| Worry about children being anxious, children not knowing what to do, leading to tensions and bad behaviour ^a^ | 1.45  (1.33 to 1.58) | 0.000 | 1.48  (1.34 to 1.62) | 0.000 | 1.30  (1.05 to 1.61) | 0.018 | 1.47  (1.29 to 1.68) | 0.000 | 1.48  (1.30 to 1.67) | 0.000 | 1.53  (1.11 to 2.09) | 0.009 |
| Worry about not being able to go out of the house nor visit loved ones ^a^ | 1.39  (1.31 to 1.48) | 0.000 | 1.38  (1.29 to 1.48) | 0.000 | 1.44  (1.24 to 1.67) | 0.000 | 1.31  (1.20 to 1.44) | 0.000 | 1.43  (1.30 to 1.58) | 0.000 | 1.65  (1.28 to 2.15) | 0.000 |
| Worry about the uncertainty of when and how normality will return ^a^ | 1.59  (1.49 to 1.70) | 0.000 | 1.62  (1.51 to 1.74) | 0.000 | 1.58  (1.36 to 1.83) | 0.000 | 1.59  (1.45 to 1.74) | 0.000 | 1.52  (1.38 to 1.68) | 0.000 | 2.29  (1.75 to 3.01) | 0.000 |
| Worry about the impact the situation will have in the community (e.g., own neighborhood) ^a^ | 1.03  (0.96 to 1.10) | 0.388 | 1.02  (0.95 to 1.09) | 0.648 | 1.05  (0.90 to 1.22) | 0.544 | 1.07  (0.97 to 1.18) | 0.157 | 1.02  (0.93 to 1.12) | 0.645 | 0.78  (0.61 to 1.01) | 0.063 |
| Worry about problems living at home ^a^ | 1.80  (1.63 to 1.99) | 0.000 | 1.70  (1.52 to 1.91) | 0.000 | 2.29  (1.81 to 2.89) | 0.000 | 1.65  (1.43 to 1.90) | 0.000 | 1.81  (1.55 to 2.11) | 0.000 | 2.99  (1.87 to 4.80) | 0.000 |
| Worry about violence at home^a^ | 0.96  (0.83 to 1.11) | 0.541 | 1.00  (0.85 to 1.18) | 0.990 | 0.83  (0.60 to 1.14) | 0.256 | 1.00  (0.81 to 1.23) | 0.967 | 0.87  (0.70 to 1.08) | 0.214 | 1.10  (0.60 to 2.02) | 0.752 |
| Follow a routine^b^ | 0.58  (0.55 to 0.62) | 0.000 | 0.60  (0.56 to 0.64) | 0.000 | 0.52  (0.45 to 0.59) | 0.000 | 0.59  (0.54 to 0.65) | 0.000 | 0.56  (0.51 to 0.61) | 0.000 | 0.70  (0.55 to 0.89) | 0.004 |
| Talk with family or friends via phone or videoconference^b^ | 0.90  (0.85 to 0.96) | 0.001 | 0.90  (0.84 to 0.97) | 0.004 | 0.88  (0.77 to 1.01) | 0.065 | 0.87  (0.80 to 0.95) | 0.002 | 0.94  (0.86 to 1.03) | 0.184 | 0.97  (0.76 to 1.25) | 0.838 |
| Watch news about Covid-19 very often (television, social media, etc)^b^ | 1.04  (0.98 to 1.11) | 0.183 | 1.03  (0.96 to 1.10) | 0.448 | 1.12  (0.97 to 1.29) | 0.117 | 1.04  (0.95 to 1.14) | 0.373 | 1.09  (0.99 to 1.20) | 0.066 | 1.05  (0.81 to 1.36) | 0.724 |
| Take the opportunity to do housework (e.g., DIY projects, cooking, etc)^b^ | 0.80  (0.75 to 0.86) | 0.000 | 0.82  (0.77 to 0.89) | 0.000 | 0.68  (0.57 to 0.80) | 0.000 | 0.84  (0.76 to 0.92) | 0.000 | 0.77  (0.70 to 0.86) | 0.000 | 0.74  (0.57 to 0.97) | 0.031 |
| Spend time outdoors (patio, balcony) or looking outside (street, sky, etc.)^b^ | 0.96  (0.91 to 1.03) | 0.261 | 0.94  (0.88 to 1.01) | 0.098 | 1.07  (0.92 to 1.23) | 0.371 | 1.03  (0.95 to 1.13) | 0.465 | 0.93  (0.84 to 1.02) | 0.102 | 0.82  (0.64 to 1.06) | 0.130 |
| Undertake relaxing activities (e.g., listen to music)^b^ | 0.78  (0.73 to 0.84) | 0.000 | 0.76  (0.70 to 0.82) | 0.000 | 0.88  (0.75 to 1.02) | 0.098 | 0.82  (0.74 to 0.91) | 0.000 | 0.74  (0.66 to 0.82) | 0.000 | 0.71  (0.54 to 0.93) | 0.013 |
| Dedicate time to oneself^b^ | 0.60  (0.54 to 0.67) | 0.000 | 0.61  (0.55 to 0.69) | 0.000 | 0.57  (0.44 to 0.73) | 0.000 | 0.68  (0.59 to 0.80) | 0.000 | 0.54  (0.46 to 0.63) | 0.000 | 0.52  (0.37 to 0.72) | 0.000 |
| Spend more time doing activities with family^b^ | 0.70  (0.65 to 0.76) | 0.000 | 0.72  (0.67 to 0.78) | 0.000 | 0.61  (0.52 to 0.73) | 0.000 | 0.74  (0.66 to 0.81) | 0.000 | 0.71  (0.63 to 0.79) | 0.000 | 0.65  (0.47 to 0.90) | 0.008 |
| Take the opportunity to learn new things (new studies, hobbies, etc) ^b^ | 0.88  (0.78 to 0.99) | 0.032 | 0.90  (0.79 to 1.03) | 0.139 | 0.80  (0.62 to 1.03) | 0.084 | 0.93  (0.79 to 1.10) | 0.421 | 0.85  (0.71 to 1.02) | 0.089 | 0.51  (0.29 to 0.90) | 0.019 |
| Take the opportunity to advance paperwork or other delayed activities ^b^ | 0.94  (0.86 to 1.03) | 0.167 | 0.92  (0.83 to 1.01) | 0.086 | 1.08  (0.89 to 1.31) | 0.449 | 0.88  (0.78 to 0.99) | 0.034 | 0.97  (0.85 to 1.11) | 0.701 | 1.43  (0.93 to 2.20) | 0.103 |
| Does not eat more to cope with situation^c^ | 0.63  (0.59 to 0.67) | 0.000 | 0.63  (0.59 to 0.68) | 0.000 | 0.61  (0.53 to 0.71) | 0.000 | 0.66  (0.60 to 0.73) | 0.000 | 0.61  (0.55 to 0.67) | 0.000 | 0.63  (0.49 to 0.80) | 0.000 |
| Does not drink more alcohol to cope with the situation^c^ | 0.78  (0.73 to 0.83) | 0.000 | 0.80  (0.75 to 0.86) | 0.000 | 0.71  (0.61 to 0.81) | 0.000 | 0.82  (0.75 to 0.90) | 0.000 | 0.75  (0.68 to 0.82) | 0.000 | 0.71  (0.53 to 0.96) | 0.028 |
| Has a job that increases risk of COVID-19^d^ | 1.03  (0.93 to 1.13) | 0.596 | 1.02  (0.92 to 1.14) | 0.717 | 1.03  (0.84 to 1.26) | 0.762 | 0.96  (0.85 to 1.10) | 0.582 | 1.09  (0.95 to 1.25) | 0.229 | 1.08  (0.34 to 3.39) | 0.901 |
| Suffer from chronic diseases that increase own risk of COVID-19 infection^d^ | 1.56  (1.44 to 1.68) | 0.000 | 1.58  (1.44 to 1.72) | 0.000 | 1.56  (1.31 to 1.85) | 0.000 | 1.49  (1.30 to 1.70) | 0.000 | 1.55  (1.40 to 1.73) | 0.000 | 2.02  (1.57 to 2.61) | 0.000 |
| Have had COVID-19 with mild symptomatology ^d^ | 1.33  (1.21 to 1.45) | 0.000 | 1.28  (1.16 to 1.42) | 0.000 | 1.52  (1.24 to 1.86) | 0.000 | 1.22  (1.07 to 1.38) | 0.003 | 1.40  (1.22 to 1.60) | 0.000 | 1.95  (1.30 to 2.92) | 0.001 |
| Have had COVID-19 with severe symptomatology ^d^ | 1.55  (1.18 to 2.03) | 0.001 | 1.58  (1.15 to 2.17) | 0.005 | 1.40  (0.82 to 2.39) | 0.215 | 1.23  (0.78 to 1.93) | 0.369 | 1.83  (1.27 to 2.65) | 0.001 | 2.35  (0.99 to 5.57) | 0.052 |
| Have family or friends with risk factors for COVID-19 (e.g., older age, previous diseases) ^d^ | 1.08  (1.02 to 1.15) | 0.012 | 1.12  (1.04 to 1.19) | 0.001 | 0.94  (0.82 to 1.08) | 0.397 | 1.17  (1.07 to 1.27) | 0.000 | 1.00  (0.91 to 1.09) | 0.994 | 0.81  (0.62 to 1.06) | 0.120 |
| Have family or friends with COVID-19 but not hospitalised ^d^ | 1.01  (0.95 to 1.08) | 0.738 | 0.98  (0.91 to 1.05) | 0.535 | 1.21  (1.04 to 1.42) | 0.015 | 1.02  (0.93 to 1.11) | 0.727 | 0.97  (0.88 to 1.08) | 0.611 | 1.20  (0.89 to 1.62) | 0.235 |
| Have family or friends with COVID-19 hospitalised ^d^ | 0.99  (0.92 to 1.06) | 0.752 | 1.01  (0.93 to 1.10) | 0.786 | 0.89  (0.74 to 1.06) | 0.183 | 1.04  (0.94 to 1.16) | 0.429 | 0.98  (0.88 to 1.10) | 0.768 | 0.78  (0.57 to 1.07) | 0.125 |
| Have family or a close friend who has died because of COVID-19 ^d^ | 1.12  (1.04 to 1.20) | 0.004 | 1.10  (1.01 to 1.20) | 0.025 | 1.23  (1.03 to 1.48) | 0.026 | 1.10  (0.98 to 1.24) | 0.112 | 1.10  (0.99 to 1.23) | 0.074 | 1.25  (0.93 to 1.69) | 0.143 |
| Have children and they take up half of my time or less^d^ | 0.76  (0.70 to 0.83) | 0.000 | 0.76  (0.70 to 0.84) | 0.000 | 0.75  (0.62 to 0.91) | 0.003 | 0.53  (0.46 to 0.61) | 0.000 | 0.94  (0.84 to 1.05) | 0.253 | 1.84  (0.84 to 4.04) | 0.128 |
| Have children and they take up most of my time^d^ | 0.82  (0.75 to 0.90) | 0.000 | 0.85  (0.77 to 0.94) | 0.002 | 0.74  (0.58 to 0.95) | 0.017 | 0.70  (0.62 to 0.79) | 0.000 | 1.16  (0.99 to 1.36) | 0.072 | 0.30  (0.03 to 2.66) | 0.281 |
| Be responsible for a dependent person/s and they take up half of my time or less^d^ | 1.16  (1.02 to 1.32) | 0.020 | 1.23  (1.07 to 1.41) | 0.003 | 0.81  (0.56 to 1.17) | 0.252 | 0.94  (0.72 to 1.22) | 0.630 | 1.26  (1.08 to 1.47) | 0.003 | 1.26  (0.72 to 2.22) | 0.416 |
| Be responsible for a dependent person/s and they take up most of the time^d^ | 1.27  (1.01 to 1.60) | 0.040 | 1.38  (1.08 to 1.76) | 0.010 | 0.71  (0.34 to 1.45) | 0.344 | 0.78  (0.49 to 1.24) | 0.287 | 1.43  (1.08 to 1.90) | 0.013 | 1.83  (0.86 to 3.91) | 0.118 |
| High social support v low social support (3-point scale) | 0.54  (0.50 to 0.59) | 0.000 | 0.56  (0.51 to 0.61) | 0.000 | 0.47  (0.38 to 0.57) | 0.000 | 0.55  (0.49 to 0.62) | 0.000 | 0.54  (0.47 to 0.60) | 0.000 | 0.56  (0.40 to 0.80) | 0.001 |
| Moderate social support v low social support (3-point scale) | 0.74  (0.69 to 0.79) | 0.000 | 0.73  (0.68 to 0.79) | 0.000 | 0.81  (0.69 to 0.95) | 0.008 | 0.78  (0.71 to 0.87) | 0.000 | 0.71  (0.64 to 0.79) | 0.000 | 0.71  (0.53 to 0.94) | 0.018 |
| Spending more than 2 hours a day reading COVID-19 news or information^d^ | 1.18  (1.09 to 1.27) | 0.000 | 1.16  (1.07 to 1.26) | 0.000 | 1.23  (1.05 to 1.44) | 0.011 | 1.10  (0.98 to 1.24) | 0.097 | 1.22  (1.10 to 1.35) | 0.000 | 1.32  (1.02 to 1.72) | 0.036 |
| Having a balcony, terrace, or garden^d^ | 0.95  (0.87 to 1.05) | 0.318 | 0.96  (0.87 to 1.07) | 0.469 | 0.99  (0.80 to 1.23) | 0.958 | 0.96  (0.84 to 1.09) | 0.482 | 0.95  (0.82 to 1.10) | 0.466 | 0.91  (0.58 to 1.44) | 0.697 |
| Weeks in confinement (9+ versus <9) | 1.13  (1.00 to 1.29) | 0.058 | 1.05  (0.91 to 1.22) | 0.513 | 1.59  (1.20 to 2.11) | 0.001 | 1.00  (0.83 to 1.22) | 0.983 | 1.19  (0.98 to 1.45) | 0.074 | 1.36  (0.86 to 2.17) | 0.191 |
| Number of people in house (5+ versus <5) | 1.01  (0.92 to 1.11) | 0.837 | 1.02  (0.92 to 1.14) | 0.696 | 0.96  (0.76 to 1.21) | 0.713 | 1.13  (0.99 to 1.29) | 0.065 | 0.89  (0.77 to 1.03) | 0.119 | 0.95  (0.48 to 1.89) | 0.888 |
| Number of rooms in house (4+ versus <4) | 1.07  (1.01 to 1.13) | 0.033 | 1.05  (0.99 to 1.13) | 0.115 | 1.12  (0.97 to 1.28) | 0.115 | 1.01  (0.92 to 1.10) | 0.858 | 1.10  (1.01 to 1.20) | 0.026 | 1.00  (0.79 to 1.28) | 0.984 |
| Being a woman versus not | 1.64  (1.52 to 1.76) | 0.000 | **-** | **-** | **-** | **-** | 1.32  (1.19 to 1.47) | 0.000 | 1.99  (1.78 to 2.22) | 0.000 | 2.12  (1.62 to 2.76) | 0.000 |
| Primary and secondary education versus postgraduate education (3-point scale) | 1.09  (1.00 to 1.18) | 0.051 | 1.04  (0.95 to 1.14) | 0.380 | 1.34  (1.11 to 1.61) | 0.002 | 0.98  (0.88 to 1.10) | 0.770 | 1.17  (1.03 to 1.33) | 0.016 | 1.02  (0.68 to 1.53) | 0.933 |
| University education versus postgraduate education (3-point scale) | 1.02  (0.95 to 1.09) | 0.534 | 1.00  (0.93 to 1.08) | 0.924 | 1.11  (0.95 to 1.31) | 0.181 | 0.97  (0.88 to 1.08) | 0.581 | 1.05  (0.95 to 1.16) | 0.315 | 1.01  (0.78 to 1.31) | 0.929 |
| Aged 65+ versus aged 15-44 years (3-point scale) | 0.45  (0.37 to 0.54) | 0.000 | 0.49  (0.39 to 0.61) | 0.000 | 0.35  (0.24 to 0.50) | 0.000 | **-** | **-** | **-** | **-** | **-** | **-** |
| Aged 45-64 versus aged 15-44 years (3-point scale) | 0.63  (0.59 to 0.67) | 0.000 | 0.67  (0.62 to 0.73) | 0.000 | 0.47  (0.40 to 0.55) | 0.000 | **-** | **-** | **-** | **-** | **-** | **-** |
| Own job future is very likely to get worse^e^ | 1.60  (1.49 to 1.72) | 0.000 | 1.58  (1.45 to 1.71) | 0.000 | 1.78  (1.51 to 2.09) | 0.000 | 1.43  (1.29 to 1.59) | 0.000 | 1.80  (1.61 to 2.01) | 0.000 | 1.65  (1.13 to 2.41) | 0.010 |
| Worried about economic consequences of COVID-19^f^ | 1.31  (1.23 to 1.41) | 0.000 | 1.32  (1.22 to 1.42) | 0.000 | 1.28  (1.08 to 1.50) | 0.004 | 1.31  (1.18 to 1.45) | 0.000 | 1.24  (1.13 to 1.37) | 0.000 | 2.02  (1.56 to 2.61) | 0.000 |
| Being a health worker versus not | 1.05  (0.89 to 1.23) | 0.550 | 1.04  (0.87 to 1.24) | 0.643 | 1.24  (0.79 to 1.94) | 0.354 | 1.22  (0.96 to 1.56) | 0.100 | 0.91  (0.72 to 1.14) | 0.397 | 0.86  (0.21 to 3.52) | 0.839 |
| Being another front-line worker (excluding health worker) versus not | 0.83  (0.71 to 0.96) | 0.014 | 0.88  (0.74 to 1.03) | 0.108 | 0.80  (0.53 to 1.20) | 0.281 | 0.93  (0.74 to 1.17) | 0.547 | 0.74  (0.60 to 0.91) | 0.004 | 0.24  (0.03 to 2.16) | 0.202 |
| Being another worker (excluding health worker and other frontline worker) versus not | 0.91  (0.81 to 1.03) | 0.152 | 0.90  (0.78 to 1.02) | 0.107 | 1.07  (0.75 to 1.55) | 0.699 | 1.00  (0.82 to 1.22) | 0.998 | 0.82  (0.69 to 0.97) | 0.018 | 1.25  (0.50 to 3.16) | 0.634 |
| Being on sick leave versus not | 1.50  (1.26 to 1.78) | 0.000 | 1.44  (1.19 to 1.73) | 0.000 | 2.06  (1.29 to 3.27) | 0.002 | 1.17  (0.87 to 1.59) | 0.302 | 1.67  (1.35 to 2.08) | 0.000 | 1.14  (0.39 to 3.37) | 0.807 |
| Being unemployed versus not | 1.03  (0.90 to 1.17) | 0.701 | 0.98  (0.84 to 1.13) | 0.738 | 1.40  (0.95 to 2.05) | 0.087 | 1.13  (0.92 to 1.38) | 0.243 | 0.86  (0.71 to 1.04) | 0.129 | 3.56  (1.17 to 10.79) | 0.025 |
| Being a student versus not | 2.13  (1.77 to 2.56) | 0.000 | 2.09  (1.70 to 2.56) | 0.000 | 2.43  (1.51 to 3.90) | 0.000 | 1.92  (1.52 to 2.43) | 0.000 | 0.00  (0.00 to .) | 1.000 | 0.00  (0.00 to .) | 1.000 |
| Being retired versus not | 0.81  (0.67 to 0.98) | 0.032 | 0.83  (0.67 to 1.04) | 0.101 | 0.87  (0.55 to 1.38) | 0.554 | 1.38  (0.13 to 14.56) | 0.789 | 0.79  (0.61 to 1.01) | 0.057 | 1.06  (0.52 to 2.17) | 0.870 |

^a^Worry a lot (score 4 on a 4-point Likert scale from 1, not worried to 4, worried a lot) versus not score 4

^b^Almost every day (score 4 on a 4-point Likert scale from 1, not any day to 4, almost every day) versus not score 4

^c^Not any day (score 1 on a 4-point Likert scale from 1, not any day to 4, almost every day) versus not score 1

^d^Present versus absent

^e^Own job future very likely to get worse (score 5 on a 5-point Likert scale from 1, very likely to get better to 5, very likely to get worse) versus not score 5

^f^Very worried (score 10 on a 10-point Likert scale from 1, not at all worried to 10, very worried) versus not score 10

**Supplement Table 4b** Odds ratios (95% confidence intervals) and probability (p) values for associations between risk and protective variables and reported anxiety

|  | **TOTAL** | | **FOR WOMEN and MEN** | | | | **FOR THREE AGE GROUPS** | | | | | |
| --- | --- | --- | --- | --- | --- | --- | --- | --- | --- | --- | --- | --- |
|  |  | | **Women** | | **Men** | | **15-44** | | **45-64** | | **65+** | |
| **Variables** | **OR (95% CI)** | **P value** | **OR (95% CI)** | **P value** | **OR (95% CI)** | **P value** | **OR (95% CI)** | **P value** | **OR (95% CI)** | **P value** | **OR (95% CI)** | **P value** |
| Worry about being infected by Covid-19 (onself or family member)^a^ | 1.49  (1.40 to 1.58) | 0.000 | 1.51  (1.41 to 1.61) | 0.000 | 1.44  (1.26 to 1.65) | 0.000 | 1.42  (1.31 to 1.55) | 0.000 | 1.55  (1.41 to 1.69) | 0.000 | 1.66  (1.29 to 2.12) | 0.000 |
| Worry about being alone and not being able to take care of oneself ^a^ | 1.22  (1.12 to 1.32) | 0.000 | 1.25  (1.14 to 1.37) | 0.000 | 1.14  (0.93 to 1.38) | 0.199 | 1.30  (1.14 to 1.50) | 0.000 | 1.10  (0.98 to 1.23) | 0.119 | 1.56  (1.21 to 2.02) | 0.001 |
| Worry about family members who are alone ^a^ | 1.22  (1.15 to 1.29) | 0.000 | 1.20  (1.12 to 1.29) | 0.000 | 1.28  (1.12 to 1.47) | 0.000 | 1.17  (1.07 to 1.27) | 0.001 | 1.27  (1.16 to 1.38) | 0.000 | 1.25  (0.98 to 1.59) | 0.070 |
| Worry about having a health emergency and not receiving any care (oneself or family member) ^a^ | 1.14  (1.07 to 1.21) | 0.000 | 1.16  (1.08 to 1.24) | 0.000 | 1.07  (0.93 to 1.23) | 0.329 | 1.11  (1.01 to 1.21) | 0.023 | 1.20  (1.09 to 1.31) | 0.000 | 1.04  (0.81 to 1.33) | 0.768 |
| Worry about job loss ^a^ | 1.01  (0.94 to 1.09) | 0.762 | 1.01  (0.93 to 1.09) | 0.892 | 1.05  (0.89 to 1.24) | 0.537 | 1.01  (0.91 to 1.12) | 0.822 | 1.03  (0.92 to 1.14) | 0.610 | 1.02  (0.67 to 1.56) | 0.920 |
| Worry about economic difficulties to buy food, pay rent, electricity etc. ^a^ | 1.12  (1.04 to 1.21) | 0.003 | 1.14  (1.05 to 1.24) | 0.002 | 1.08  (0.91 to 1.28) | 0.362 | 1.19  (1.07 to 1.32) | 0.002 | 1.10  (0.99 to 1.23) | 0.086 | 0.78  (0.56 to 1.09) | 0.150 |
| Worry about loss of an academic year or the educational future of own children ^a^ | 0.94  (0.87 to 1.02) | 0.131 | 0.98  (0.90 to 1.07) | 0.674 | 0.82  (0.69 to 0.98) | 0.028 | 1.15  (1.02 to 1.29) | 0.022 | 0.81  (0.73 to 0.90) | 0.000 | 0.93  (0.64 to 1.34) | 0.686 |
| Worry about inability to combine having to work from home while taking care of family (children, dependent persons, etc) ^a^ | 1.30  (1.20 to 1.41) | 0.000 | 1.34  (1.23 to 1.46) | 0.000 | 1.10  (0.91 to 1.34) | 0.319 | 1.28  (1.15 to 1.43) | 0.000 | 1.39  (1.23 to 1.56) | 0.000 | 0.93  (0.57 to 1.52) | 0.770 |
| Worry about children being anxious, children not knowing what to do, leading to tensions and bad behaviour ^a^ | 1.62  (1.49 to 1.76) | 0.000 | 1.61  (1.47 to 1.76) | 0.000 | 1.72  (1.43 to 2.08) | 0.000 | 1.57  (1.39 to 1.78) | 0.000 | 1.68  (1.49 to 1.88) | 0.000 | 1.90  (1.44 to 2.51) | 0.000 |
| Worry about not being able to go out of the house nor visit loved ones ^a^ | 1.38  (1.30 to 1.46) | 0.000 | 1.36  (1.27 to 1.46) | 0.000 | 1.42  (1.24 to 1.63) | 0.000 | 1.37  (1.25 to 1.50) | 0.000 | 1.34  (1.23 to 1.47) | 0.000 | 1.58  (1.26 to 1.98) | 0.000 |
| Worry about the uncertainty of when and how normality will return ^a^ | 1.89  (1.78 to 2.01) | 0.000 | 1.87  (1.75 to 2.00) | 0.000 | 2.06  (1.79 to 2.36) | 0.000 | 1.90  (1.73 to 2.07) | 0.000 | 1.81  (1.65 to 1.98) | 0.000 | 2.38  (1.88 to 3.02) | 0.000 |
| Worry about the impact the situation will have in the community (e.g., own neighborhood) ^a^ | 1.07  (1.00 to 1.14) | 0.036 | 1.05  (0.98 to 1.13) | 0.160 | 1.12  (0.98 to 1.29) | 0.101 | 1.06  (0.97 to 1.17) | 0.186 | 1.07  (0.98 to 1.17) | 0.111 | 1.07  (0.85 to 1.34) | 0.563 |
| Worry about problems living at home ^a^ | 2.05  (1.86 to 2.27) | 0.000 | 1.95  (1.74 to 2.18) | 0.000 | 2.58  (2.07 to 3.20) | 0.000 | 2.08  (1.80 to 2.40) | 0.000 | 1.75  (1.51 to 2.03) | 0.000 | 6.06  (3.84 to 9.55) | 0.000 |
| Worry about violence at home^a^ | 0.88  (0.76 to 1.01) | 0.072 | 0.89  (0.76 to 1.06) | 0.184 | 0.83  (0.62 to 1.11) | 0.201 | 0.90  (0.73 to 1.11) | 0.315 | 0.89  (0.72 to 1.10) | 0.291 | 0.62  (0.34 to 1.13) | 0.117 |
| Follow a routine^b^ | 0.80  (0.75 to 0.85) | 0.000 | 0.82  (0.77 to 0.87) | 0.000 | 0.73  (0.64 to 0.83) | 0.000 | 0.79  (0.72 to 0.86) | 0.000 | 0.82  (0.75 to 0.89) | 0.000 | 0.79  (0.63 to 0.98) | 0.030 |
| Talk with family or friends via phone or videoconference^b^ | 0.92  (0.86 to 0.97) | 0.004 | 0.90  (0.84 to 0.96) | 0.002 | 0.99  (0.87 to 1.13) | 0.876 | 0.86  (0.79 to 0.94) | 0.001 | 0.97  (0.89 to 1.06) | 0.454 | 1.02  (0.81 to 1.28) | 0.899 |
| Watch news about Covid-19 very often (television, social media, etc)^b^ | 1.12  (1.05 to 1.19) | 0.000 | 1.11  (1.04 to 1.18) | 0.002 | 1.17  (1.02 to 1.33) | 0.026 | 1.10  (1.01 to 1.20) | 0.027 | 1.16  (1.06 to 1.27) | 0.001 | 1.15  (0.91 to 1.46) | 0.242 |
| Take the opportunity to do housework (e.g., DIY projects, cooking, etc)^b^ | 0.89  (0.83 to 0.95) | 0.000 | 0.90  (0.84 to 0.96) | 0.003 | 0.84  (0.72 to 0.98) | 0.024 | 0.86  (0.78 to 0.94) | 0.002 | 0.90  (0.82 to 0.99) | 0.024 | 0.98  (0.78 to 1.23) | 0.856 |
| Spend time outdoors (patio, balcony) or looking outside (street, sky, etc.)^b^ | 0.95  (0.90 to 1.01) | 0.094 | 0.93  (0.87 to 0.99) | 0.025 | 1.04  (0.91 to 1.20) | 0.530 | 1.01  (0.93 to 1.10) | 0.799 | 0.90  (0.82 to 0.98) | 0.017 | 0.99  (0.79 to 1.25) | 0.937 |
| Undertake relaxing activities (e.g., listen to music)^b^ | 0.72  (0.68 to 0.77) | 0.000 | 0.72  (0.66 to 0.77) | 0.000 | 0.74  (0.63 to 0.85) | 0.000 | 0.73  (0.66 to 0.81) | 0.000 | 0.73  (0.66 to 0.81) | 0.000 | 0.63  (0.49 to 0.79) | 0.000 |
| Dedicate time to oneself^b^ | 0.69  (0.63 to 0.76) | 0.000 | 0.71  (0.64 to 0.78) | 0.000 | 0.62  (0.50 to 0.78) | 0.000 | 0.68  (0.59 to 0.79) | 0.000 | 0.65  (0.57 to 0.75) | 0.000 | 0.80  (0.61 to 1.04) | 0.094 |
| Spend more time doing activities with family^b^ | 0.73  (0.68 to 0.78) | 0.000 | 0.74  (0.69 to 0.80) | 0.000 | 0.70  (0.60 to 0.82) | 0.000 | 0.75  (0.68 to 0.82) | 0.000 | 0.72  (0.65 to 0.80) | 0.000 | 0.81  (0.63 to 1.06) | 0.120 |
| Take the opportunity to learn new things (new studies, hobbies, etc) ^b^ | 0.84  (0.76 to 0.94) | 0.003 | 0.83  (0.73 to 0.94) | 0.004 | 0.89  (0.70 to 1.11) | 0.296 | 0.95  (0.81 to 1.12) | 0.528 | 0.77  (0.65 to 0.91) | 0.002 | 0.78  (0.52 to 1.17) | 0.233 |
| Take the opportunity to advance paperwork or other delayed activities ^b^ | 1.05  (0.96 to 1.13) | 0.284 | 1.04  (0.94 to 1.14) | 0.451 | 1.13  (0.95 to 1.35) | 0.179 | 0.96  (0.85 to 1.08) | 0.490 | 1.11  (0.98 to 1.25) | 0.103 | 1.29  (0.90 to 1.85) | 0.164 |
| Does not eat more to cope with situation^c^ | 0.88  (0.83 to 0.93) | 0.000 | 0.88  (0.82 to 0.94) | 0.000 | 0.89  (0.78 to 1.01) | 0.079 | 0.89  (0.81 to 0.97) | 0.011 | 0.88  (0.81 to 0.97) | 0.006 | 0.80  (0.65 to 1.00) | 0.046 |
| Does not drink more alcohol to cope with the situation^c^ | 0.83  (0.78 to 0.88) | 0.000 | 0.84  (0.79 to 0.91) | 0.000 | 0.80  (0.70 to 0.92) | 0.001 | 0.83  (0.76 to 0.91) | 0.000 | 0.85  (0.77 to 0.93) | 0.000 | 0.77  (0.58 to 1.03) | 0.078 |
| Has a job that increases risk of COVID-19^d^ | 1.10  (1.01 to 1.20) | 0.036 | 1.12  (1.01 to 1.24) | 0.033 | 1.04  (0.87 to 1.26) | 0.662 | 1.01  (0.89 to 1.15) | 0.876 | 1.21  (1.06 to 1.37) | 0.004 | 0.76  (0.27 to 2.14) | 0.605 |
| Suffer from chronic diseases that increase own risk of COVID-19 infection^d^ | 1.36  (1.26 to 1.47) | 0.000 | 1.33  (1.22 to 1.46) | 0.000 | 1.50  (1.28 to 1.76) | 0.000 | 1.36  (1.19 to 1.55) | 0.000 | 1.35  (1.22 to 1.50) | 0.000 | 1.43  (1.13 to 1.82) | 0.003 |
| Have had COVID-19 with mild symptomatology ^d^ | 1.24  (1.14 to 1.36) | 0.000 | 1.19  (1.08 to 1.32) | 0.001 | 1.44  (1.19 to 1.75) | 0.000 | 1.28  (1.12 to 1.45) | 0.000 | 1.15  (1.01 to 1.31) | 0.036 | 1.67  (1.14 to 2.45) | 0.008 |
| Have had COVID-19 with severe symptomatology ^d^ | 1.42  (1.09 to 1.85) | 0.009 | 1.41  (1.03 to 1.93) | 0.031 | 1.37  (0.83 to 2.28) | 0.219 | 1.44  (0.93 to 2.23) | 0.105 | 1.40  (0.97 to 2.01) | 0.069 | 2.10  (0.91 to 4.80) | 0.081 |
| Have family or friends with risk factors for COVID-19 (e.g., older age, previous diseases) ^d^ | 1.13  (1.07 to 1.20) | 0.000 | 1.14  (1.07 to 1.22) | 0.000 | 1.07  (0.94 to 1.22) | 0.282 | 1.29  (1.18 to 1.40) | 0.000 | 1.02  (0.93 to 1.11) | 0.720 | 0.87  (0.68 to 1.10) | 0.247 |
| Have family or friends with COVID-19 but not hospitalised ^d^ | 0.99  (0.93 to 1.06) | 0.833 | 1.00  (0.94 to 1.08) | 0.935 | 0.96  (0.83 to 1.12) | 0.639 | 0.96  (0.88 to 1.05) | 0.347 | 1.02  (0.93 to 1.12) | 0.667 | 1.00  (0.76 to 1.32) | 0.998 |
| Have family or friends with COVID-19 hospitalised ^d^ | 1.05  (0.98 to 1.12) | 0.202 | 1.05  (0.98 to 1.14) | 0.178 | 1.02  (0.87 to 1.21) | 0.777 | 1.10  (1.00 to 1.22) | 0.062 | 1.01  (0.91 to 1.12) | 0.807 | 1.09  (0.83 to 1.44) | 0.518 |
| Have family or a close friend who has died because of COVID-19 ^d^ | 1.10  (1.03 to 1.19) | 0.007 | 1.08  (1.00 to 1.17) | 0.062 | 1.22  (1.03 to 1.44) | 0.024 | 0.98  (0.88 to 1.10) | 0.785 | 1.21  (1.09 to 1.33) | 0.000 | 1.06  (0.81 to 1.40) | 0.655 |
| Have children and they take up half of my time or less^d^ | 1.00  (0.93 to 1.09) | 0.902 | 0.97  (0.89 to 1.06) | 0.562 | 1.16  (0.98 to 1.37) | 0.087 | 0.89  (0.78 to 1.02) | 0.086 | 1.09  (0.98 to 1.20) | 0.100 | 1.92  (0.92 to 3.99) | 0.082 |
| Have children and they take up most of my time^d^ | 1.10  (1.01 to 1.20) | 0.036 | 1.11  (1.01 to 1.22) | 0.036 | 1.12  (0.90 to 1.39) | 0.297 | 1.00  (0.89 to 1.12) | 0.960 | 1.38  (1.19 to 1.61) | 0.000 | 1.13  (0.27 to 4.72) | 0.872 |
| Be responsible for a dependent person/s and they take up half of my time or less^d^ | 1.15  (1.02 to 1.30) | 0.023 | 1.20  (1.05 to 1.37) | 0.007 | 0.84  (0.59 to 1.18) | 0.310 | 1.17  (0.91 to 1.51) | 0.226 | 1.16  (1.00 to 1.35) | 0.048 | 1.11  (0.65 to 1.88) | 0.706 |
| Be responsible for a dependent person/s and they take up most of the time^d^ | 1.61  (1.30 to 2.00) | 0.000 | 1.61  (1.27 to 2.03) | 0.000 | 1.37  (0.75 to 2.49) | 0.302 | 1.38  (0.89 to 2.14) | 0.144 | 1.70  (1.30 to 2.22) | 0.000 | 1.59  (0.78 to 3.24) | 0.206 |
| High social support v low social support (3-point scale) | 0.65  (0.60 to 0.71) | 0.000 | 0.68  (0.62 to 0.74) | 0.000 | 0.57  (0.48 to 0.69) | 0.000 | 0.64  (0.57 to 0.72) | 0.000 | 0.65  (0.58 to 0.73) | 0.000 | 0.75  (0.55 to 1.03) | 0.079 |
| Moderate social support v low social support (3-point scale) | 0.80  (0.75 to 0.86) | 0.000 | 0.80  (0.74 to 0.87) | 0.000 | 0.81  (0.70 to 0.94) | 0.005 | 0.78  (0.71 to 0.87) | 0.000 | 0.79  (0.72 to 0.88) | 0.000 | 1.03  (0.78 to 1.36) | 0.818 |
| Spending more than 2 hours a day reading COVID-19 news or information^d^ | 1.32  (1.23 to 1.41) | 0.000 | 1.30  (1.20 to 1.41) | 0.000 | 1.36  (1.17 to 1.57) | 0.000 | 1.33  (1.19 to 1.49) | 0.000 | 1.27  (1.15 to 1.40) | 0.000 | 1.57  (1.25 to 1.98) | 0.000 |
| Having a balcony, terrace, or garden^d^ | 0.88  (0.81 to 0.97) | 0.007 | 0.92  (0.83 to 1.02) | 0.100 | 0.79  (0.65 to 0.96) | 0.020 | 0.93  (0.82 to 1.06) | 0.262 | 0.86  (0.75 to 0.98) | 0.027 | 0.69  (0.47 to 1.01) | 0.059 |
| Weeks in confinement (9+ versus <9) | 1.06  (0.93 to 1.20) | 0.401 | 1.03  (0.89 to 1.19) | 0.683 | 1.22  (0.92 to 1.62) | 0.167 | 1.02  (0.84 to 1.24) | 0.814 | 1.01  (0.83 to 1.22) | 0.945 | 1.44  (0.94 to 2.21) | 0.093 |
| Number of people in house (5+ versus <5) | 1.04  (0.95 to 1.14) | 0.399 | 1.05  (0.95 to 1.16) | 0.353 | 0.99  (0.80 to 1.21) | 0.889 | 1.02  (0.90 to 1.16) | 0.758 | 1.05  (0.92 to 1.20) | 0.494 | 1.02  (0.58 to 1.83) | 0.933 |
| Number of rooms in house (4+ versus <4) | 1.05  (0.99 to 1.11) | 0.098 | 1.06  (0.99 to 1.13) | 0.075 | 1.01  (0.89 to 1.15) | 0.850 | 0.99  (0.91 to 1.07) | 0.791 | 1.10  (1.01 to 1.20) | 0.022 | 1.06  (0.86 to 1.32) | 0.579 |
| Being a woman versus not | 1.57  (1.47 to 1.69) | 0.000 |  |  |  |  | 1.34  (1.20 to 1.49) | 0.000 | 1.77  (1.60 to 1.96) | 0.000 | 1.92  (1.52 to 2.42) | 0.000 |
| Primary and secondary education versus postgraduate education (3-point scale) | 0.93  (0.86 to 1.00) | 0.065 | 0.91  (0.83 to 0.99) | 0.037 | 1.05  (0.88 to 1.26) | 0.565 | 0.94  (0.84 to 1.05) | 0.248 | 0.99  (0.88 to 1.12) | 0.879 | 0.60  (0.41 to 0.89) | 0.011 |
| University education versus postgraduate education (3-point scale) | 0.93  (0.87 to 0.99) | 0.021 | 0.92  (0.85 to 0.99) | 0.022 | 0.96  (0.83 to 1.11) | 0.589 | 1.01  (0.91 to 1.11) | 0.911 | 0.92  (0.83 to 1.01) | 0.073 | 0.68  (0.54 to 0.85) | 0.001 |
| Aged 65+ versus aged 15-44 years (3-point scale) | 0.61  (0.51 to 0.72) | 0.000 | 0.67  (0.55 to 0.83) | 0.000 | 0.45  (0.32 to 0.63) | 0.000 |  |  |  |  |  |  |
| Aged 45-64 versus aged 15-44 years (3-point scale) | 0.59  (0.55 to 0.63) | 0.000 | 0.63  (0.58 to 0.68) | 0.000 | 0.46  (0.40 to 0.53) | 0.000 |  |  |  |  |  |  |
| Own job future is very likely to get worse^e^ | 1.49  (1.39 to 1.60) | 0.000 | 1.49  (1.38 to 1.62) | 0.000 | 1.54  (1.32 to 1.80) | 0.000 | 1.33  (1.20 to 1.48) | 0.000 | 1.66  (1.50 to 1.85) | 0.000 | 1.48  (1.04 to 2.10) | 0.030 |
| Worried about economic consequences of COVID-19^f^ | 1.46  (1.37 to 1.56) | 0.000 | 1.49  (1.38 to 1.60) | 0.000 | 1.29  (1.11 to 1.50) | 0.001 | 1.40  (1.27 to 1.55) | 0.000 | 1.50  (1.37 to 1.64) | 0.000 | 1.49  (1.18 to 1.88) | 0.001 |
| Being a health worker versus not | 1.38  (1.18 to 1.62) | 0.000 | 1.34  (1.13 to 1.59) | 0.001 | 1.76  (1.16 to 2.67) | 0.008 | 1.45  (1.15 to 1.84) | 0.002 | 1.26  (1.02 to 1.56) | 0.036 | 1.17  (0.31 to 4.46) | 0.814 |
| Being another front-line worker (excluding health worker) versus not | 1.03  (0.90 to 1.19) | 0.640 | 1.03  (0.88 to 1.20) | 0.738 | 1.15  (0.79 to 1.68) | 0.455 | 1.09  (0.87 to 1.35) | 0.459 | 0.92  (0.76 to 1.12) | 0.411 | 3.63  (1.16 to 11.39) | 0.027 |
| Being another worker (excluding health worker and other frontline worker) versus not | 1.07  (0.94 to 1.21) | 0.298 | 1.07  (0.94 to 1.23) | 0.286 | 1.10  (0.78 to 1.56) | 0.590 | 1.08  (0.89 to 1.30) | 0.455 | 0.98  (0.83 to 1.16) | 0.824 | 1.70  (0.71 to 4.04) | 0.231 |
| Being on sick leave versus not | 1.28  (1.08 to 1.53) | 0.004 | 1.22  (1.01 to 1.47) | 0.037 | 1.82  (1.16 to 2.85) | 0.009 | 0.99  (0.73 to 1.34) | 0.953 | 1.40  (1.12 to 1.73) | 0.003 | 2.45  (0.88 to 6.85) | 0.088 |
| Being unemployed versus not | 1.11  (0.97 to 1.26) | 0.131 | 1.10  (0.95 to 1.27) | 0.190 | 1.19  (0.83 to 1.72) | 0.346 | 1.15  (0.95 to 1.41) | 0.158 | 0.97  (0.81 to 1.17) | 0.766 | 2.07  (0.68 to 6.32) | 0.202 |
| Being a student versus not | 1.70  (1.41 to 2.05) | 0.000 | 1.63  (1.32 to 2.00) | 0.000 | 1.98  (1.24 to 3.16) | 0.004 | 1.53  (1.20 to 1.93) | 0.000 | 4.63  (0.45 to 48.00) | 0.199 | 1E+012  (0.00 to .) | 1.000 |
| Being retired versus not | 0.79  (0.65 to 0.95) | 0.013 | 0.78  (0.63 to 0.97) | 0.028 | 0.87  (0.56 to 1.35) | 0.537 | 2.03  (0.19 to 21.43) | 0.556 | 0.70  (0.54 to 0.89) | 0.004 | 1.33  (0.67 to 2.63) | 0.420 |

^a^Worry a lot (score 4 on a 4-point Likert scale from 1, not worried to 4, worried a lot) versus not score 4

^b^Almost every day (score 4 on a 4-point Likert scale from 1, not any day to 4, almost every day) versus not score 4

^c^Not any day (score 1 on a 4-point Likert scale from 1, not any day to 4, almost every day) versus not score 1

^d^Present versus absent

^e^Own job future very likely to get worse (score 5 on a 5-point Likert scale from 1, very likely to get better to 5, very likely to get worse) versus not score 5

^f^Very worried (score 10 on a 10-point Likert scale from 1, not at all worried to 10, very worried) versus not score 10

**Supplement Table 4c** Odds ratios (95% confidence intervals) and probability (p) values for associations between risk and protective variables and reported lack of mental well-being

|  | **TOTAL** | | **FOR WOMEN and MEN** | | | | **FOR THREE AGE GROUPS** | | | | | |
| --- | --- | --- | --- | --- | --- | --- | --- | --- | --- | --- | --- | --- |
|  |  | | **Women** | | **Men** | | **15-44** | | **45-64** | | **65+** | |
| **Variables** | **OR (95% CI)** | **P value** | **OR (95% CI)** | **P value** | **OR (95% CI)** | **P value** | **OR (95% CI)** | **P value** | **OR (95% CI)** | **P value** | **OR (95% CI)** | **P value** |
| Worry about being infected by Covid-19 (onself or family member)^a^ | 1.16 (1.10 to 1.24) | 0.000 | 1.17 (1.09 to 1.26) | 0.000 | 1.15 (1.03 to 1.28) | 0.013 | 1.27 (1.13 to 1.42) | 0.000 | 1.12 (1.03 to 1.21) | 0.008 | 1.22 (1.05 to 1.42) | 0.011 |
| Worry about being alone and not being able to take care of oneself ^a^ | 1.11 (1.01 to 1.23) | 0.033 | 1.14 (1.01 to 1.28) | 0.034 | 1.07 (0.89 to 1.27) | 0.481 | 1.21 (0.96 to 1.52) | 0.099 | 1.04 (0.91 to 1.18) | 0.558 | 1.29 (1.04 to 1.61) | 0.023 |
| Worry about family members who are alone ^a^ | 1.12 (1.05 to 1.19) | 0.001 | 1.13 (1.05 to 1.22) | 0.002 | 1.09 (0.97 to 1.22) | 0.168 | 1.09 (0.97 to 1.23) | 0.147 | 1.13 (1.04 to 1.23) | 0.003 | 1.14 (0.95 to 1.37) | 0.162 |
| Worry about having a health emergency and not receiving any care (oneself or family member) ^a^ | 0.93 (0.88 to 0.99) | 0.026 | 0.95 (0.88 to 1.02) | 0.172 | 0.89 (0.80 to 0.99) | 0.035 | 0.89 (0.80 to 1.00) | 0.041 | 0.98 (0.90 to 1.06) | 0.650 | 0.84 (0.72 to 0.98) | 0.030 |
| Worry about job loss ^a^ | 0.93 (0.86 to 1.01) | 0.073 | 0.92 (0.83 to 1.01) | 0.087 | 0.96 (0.83 to 1.11) | 0.587 | 0.99 (0.85 to 1.14) | 0.858 | 0.88 (0.79 to 0.98) | 0.019 | 1.18 (0.85 to 1.64) | 0.313 |
| Worry about economic difficulties to buy food, pay rent, electricity etc. ^a^ | 0.92 (0.85 to 1.00) | 0.064 | 0.90 (0.81 to 1.00) | 0.047 | 0.98 (0.84 to 1.15) | 0.831 | 0.92 (0.79 to 1.07) | 0.275 | 0.93 (0.83 to 1.05) | 0.240 | 0.90 (0.70 to 1.17) | 0.430 |
| Worry about loss of an academic year or the educational future of own children ^a^ | 0.81 (0.75 to 0.88) | 0.000 | 0.79 (0.71 to 0.87) | 0.000 | 0.87 (0.75 to 1.01) | 0.064 | 0.85 (0.72 to 1.01) | 0.065 | 0.78 (0.70 to 0.86) | 0.000 | 0.92 (0.70 to 1.23) | 0.590 |
| Worry about inability to combine having to work from home while taking care of family (children, dependent persons, etc) ^a^ | 1.23 (1.11 to 1.36) | 0.000 | 1.27 (1.13 to 1.44) | 0.000 | 1.11 (0.92 to 1.35) | 0.272 | 1.33 (1.13 to 1.56) | 0.001 | 1.28 (1.11 to 1.48) | 0.001 | 0.59 (0.39 to 0.91) | 0.016 |
| Worry about children being anxious, children not knowing what to do, leading to tensions and bad behaviour ^a^ | 1.44 (1.29 to 1.60) | 0.000 | 1.50 (1.32 to 1.71) | 0.000 | 1.29 (1.07 to 1.56) | 0.008 | 1.42 (1.17 to 1.73) | 0.000 | 1.56 (1.35 to 1.80) | 0.000 | 1.25 (0.97 to 1.61) | 0.081 |
| Worry about not being able to go out of the house nor visit loved ones ^a^ | 1.30 (1.21 to 1.39) | 0.000 | 1.35 (1.24 to 1.46) | 0.000 | 1.18 (1.04 to 1.33) | 0.008 | 1.46 (1.29 to 1.66) | 0.000 | 1.23 (1.13 to 1.35) | 0.000 | 1.22 (1.03 to 1.45) | 0.021 |
| Worry about the uncertainty of when and how normality will return ^a^ | 1.69 (1.58 to 1.81) | 0.000 | 1.79 (1.65 to 1.95) | 0.000 | 1.49 (1.32 to 1.68) | 0.000 | 1.91 (1.68 to 2.18) | 0.000 | 1.56 (1.43 to 1.71) | 0.000 | 1.79 (1.51 to 2.11) | 0.000 |
| Worry about the impact the situation will have in the community (e.g., own neighborhood) ^a^ | 1.03 (0.97 to 1.10) | 0.374 | 1.03 (0.95 to 1.11) | 0.467 | 1.02 (0.91 to 1.14) | 0.783 | 0.95 (0.84 to 1.08) | 0.458 | 1.07 (0.98 to 1.16) | 0.142 | 1.04 (0.89 to 1.21) | 0.624 |
| Worry about problems living at home ^a^ | 1.93 (1.64 to 2.27) | 0.000 | 2.20 (1.78 to 2.72) | 0.000 | 1.56 (1.20 to 2.03) | 0.001 | 2.11 (1.56 to 2.85) | 0.000 | 1.81 (1.46 to 2.24) | 0.000 | 1.84 (1.13 to 3.01) | 0.015 |
| Worry about violence at home^a^ | 0.82 (0.69 to 0.97) | 0.021 | 0.79 (0.63 to 0.99) | 0.040 | 0.86 (0.65 to 1.12) | 0.262 | 0.94 (0.67 to 1.31) | 0.694 | 0.74 (0.59 to 0.93) | 0.010 | 0.90 (0.57 to 1.42) | 0.646 |
| Follow a routine^b^ | 0.58 (0.54 to 0.61) | 0.000 | 0.54 (0.50 to 0.59) | 0.000 | 0.63 (0.57 to 0.70) | 0.000 | 0.52 (0.46 to 0.59) | 0.000 | 0.57 (0.52 to 0.62) | 0.000 | 0.70 (0.60 to 0.81) | 0.000 |
| Talk with family or friends via phone or videoconference^b^ | 0.91 (0.86 to 0.97) | 0.002 | 0.90 (0.83 to 0.97) | 0.005 | 0.94 (0.85 to 1.04) | 0.239 | 0.83 (0.74 to 0.93) | 0.001 | 0.92 (0.85 to 1.00) | 0.045 | 1.04 (0.90 to 1.21) | 0.591 |
| Watch news about Covid-19 very often (television, social media, etc)^b^ | 0.98 (0.93 to 1.04) | 0.487 | 0.98 (0.91 to 1.05) | 0.534 | 1.00 (0.90 to 1.11) | 0.989 | 1.06 (0.95 to 1.18) | 0.264 | 0.95 (0.88 to 1.03) | 0.242 | 0.99 (0.85 to 1.15) | 0.882 |
| Take the opportunity to do housework (e.g., DIY projects, cooking, etc)^b^ | 0.81 (0.76 to 0.85) | 0.000 | 0.81 (0.76 to 0.87) | 0.000 | 0.79 (0.71 to 0.89) | 0.000 | 0.84 (0.75 to 0.94) | 0.002 | 0.82 (0.76 to 0.89) | 0.000 | 0.70 (0.60 to 0.80) | 0.000 |
| Spend time outdoors (patio, balcony) or looking outside (street, sky, etc.)^b^ | 0.87 (0.82 to 0.93) | 0.000 | 0.88 (0.82 to 0.95) | 0.001 | 0.85 (0.77 to 0.95) | 0.003 | 0.91 (0.81 to 1.02) | 0.107 | 0.89 (0.82 to 0.96) | 0.004 | 0.78 (0.67 to 0.91) | 0.002 |
| Undertake relaxing activities (e.g., listen to music)^b^ | 0.71 (0.67 to 0.76) | 0.000 | 0.70 (0.65 to 0.75) | 0.000 | 0.75 (0.67 to 0.83) | 0.000 | 0.68 (0.61 to 0.76) | 0.000 | 0.71 (0.65 to 0.77) | 0.000 | 0.74 (0.64 to 0.86) | 0.000 |
| Dedicate time to oneself^b^ | 0.58 (0.54 to 0.62) | 0.000 | 0.57 (0.52 to 0.61) | 0.000 | 0.61 (0.54 to 0.70) | 0.000 | 0.58 (0.50 to 0.66) | 0.000 | 0.57 (0.52 to 0.63) | 0.000 | 0.57 (0.49 to 0.67) | 0.000 |
| Spend more time doing activities with family^b^ | 0.69 (0.65 to 0.73) | 0.000 | 0.69 (0.64 to 0.74) | 0.000 | 0.70 (0.63 to 0.78) | 0.000 | 0.67 (0.60 to 0.75) | 0.000 | 0.68 (0.63 to 0.74) | 0.000 | 0.82 (0.70 to 0.95) | 0.008 |
| Take the opportunity to learn new things (new studies, hobbies, etc) ^b^ | 0.76 (0.70 to 0.83) | 0.000 | 0.77 (0.69 to 0.85) | 0.000 | 0.77 (0.67 to 0.89) | 0.000 | 0.83 (0.71 to 0.97) | 0.022 | 0.75 (0.67 to 0.85) | 0.000 | 0.67 (0.55 to 0.82) | 0.000 |
| Take the opportunity to advance paperwork or other delayed activities ^b^ | 0.73 (0.68 to 0.79) | 0.000 | 0.73 (0.67 to 0.80) | 0.000 | 0.74 (0.65 to 0.84) | 0.000 | 0.73 (0.64 to 0.83) | 0.000 | 0.71 (0.65 to 0.78) | 0.000 | 0.87 (0.70 to 1.07) | 0.175 |
| Does not eat more to cope with situation^c^ | 0.78 (0.73 to 0.82) | 0.000 | 0.77 (0.72 to 0.82) | 0.000 | 0.80 (0.73 to 0.89) | 0.000 | 0.80 (0.72 to 0.89) | 0.000 | 0.76 (0.70 to 0.82) | 0.000 | 0.82 (0.71 to 0.94) | 0.005 |
| Does not drink more alcohol to cope with the situation^c^ | 0.79 (0.73 to 0.84) | 0.000 | 0.79 (0.72 to 0.85) | 0.000 | 0.78 (0.70 to 0.88) | 0.000 | 0.70 (0.62 to 0.79) | 0.000 | 0.85 (0.78 to 0.93) | 0.000 | 0.74 (0.60 to 0.91) | 0.005 |
| Has a job that increases risk of COVID-19^d^ | 0.89 (0.82 to 0.98) | 0.017 | 0.92 (0.82 to 1.03) | 0.144 | 0.87 (0.74 to 1.01) | 0.068 | 0.88 (0.75 to 1.02) | 0.095 | 0.88 (0.78 to 0.99) | 0.032 | 1.15 (0.60 to 2.18) | 0.671 |
| Suffer from chronic diseases that increase own risk of COVID-19 infection^d^ | 1.28 (1.18 to 1.40) | 0.000 | 1.26 (1.13 to 1.40) | 0.000 | 1.37 (1.19 to 1.57) | 0.000 | 1.23 (1.01 to 1.49) | 0.039 | 1.25 (1.12 to 1.39) | 0.000 | 1.47 (1.23 to 1.74) | 0.000 |
| Have had COVID-19 with mild symptomatology ^d^ | 1.20 (1.08 to 1.33) | 0.000 | 1.19 (1.05 to 1.34) | 0.006 | 1.23 (1.02 to 1.48) | 0.028 | 1.32 (1.10 to 1.58) | 0.003 | 1.15 (1.01 to 1.32) | 0.041 | 1.15 (0.84 to 1.56) | 0.384 |
| Have had COVID-19 with severe symptomatology ^d^ | 1.21 (0.88 to 1.67) | 0.229 | 1.02 (0.68 to 1.52) | 0.938 | 1.72 (1.02 to 2.91) | 0.042 | 0.77 (0.42 to 1.39) | 0.383 | 1.41 (0.94 to 2.11) | 0.097 | 1.63 (0.67 to 3.98) | 0.280 |
| Have family or friends with risk factors for COVID-19 (e.g., older age, previous diseases) ^d^ | 1.24 (1.17 to 1.31) | 0.000 | 1.24 (1.16 to 1.33) | 0.000 | 1.24 (1.12 to 1.37) | 0.000 | 1.29 (1.16 to 1.44) | 0.000 | 1.23 (1.14 to 1.33) | 0.000 | 1.12 (0.96 to 1.31) | 0.163 |
| Have family or friends with COVID-19 but not hospitalised ^d^ | 1.11 (1.04 to 1.19) | 0.001 | 1.14 (1.05 to 1.23) | 0.001 | 1.04 (0.92 to 1.18) | 0.496 | 1.20 (1.06 to 1.34) | 0.003 | 1.06 (0.97 to 1.16) | 0.181 | 1.13 (0.94 to 1.35) | 0.189 |
| Have family or friends with COVID-19 hospitalised ^d^ | 1.06 (0.98 to 1.14) | 0.122 | 1.08 (0.99 to 1.18) | 0.066 | 1.01 (0.88 to 1.15) | 0.921 | 0.97 (0.85 to 1.11) | 0.681 | 1.11 (1.01 to 1.22) | 0.036 | 1.09 (0.91 to 1.31) | 0.340 |
| Have family or a close friend who has died because of COVID-19 ^d^ | 0.96 (0.89 to 1.03) | 0.233 | 0.96 (0.88 to 1.05) | 0.350 | 0.97 (0.84 to 1.11) | 0.613 | 0.96 (0.83 to 1.12) | 0.622 | 0.94 (0.86 to 1.04) | 0.227 | 1.00 (0.83 to 1.20) | 0.992 |
| Have children and they take up half of my time or less^d^ | 1.03 (0.95 to 1.11) | 0.494 | 1.00 (0.91 to 1.09) | 0.948 | 1.10 (0.96 to 1.25) | 0.175 | 0.74 (0.64 to 0.86) | 0.000 | 1.14 (1.05 to 1.25) | 0.003 | 1.61 (0.89 to 2.88) | 0.112 |
| Have children and they take up most of my time^d^ | 1.16 (1.05 to 1.28) | 0.003 | 1.14 (1.02 to 1.28) | 0.025 | 1.25 (1.03 to 1.53) | 0.026 | 1.00 (0.87 to 1.16) | 0.946 | 1.32 (1.12 to 1.55) | 0.001 | 0.62 (0.19 to 1.97) | 0.416 |
| Be responsible for a dependent person/s and they take up half of my time or less^d^ | 1.02 (0.90 to 1.16) | 0.772 | 1.05 (0.90 to 1.22) | 0.559 | 0.96 (0.75 to 1.24) | 0.769 | 0.66 (0.48 to 0.92) | 0.015 | 1.03 (0.89 to 1.20) | 0.701 | 1.63 (1.12 to 2.36) | 0.011 |
| Be responsible for a dependent person/s and they take up most of the time^d^ | 0.92 (0.71 to 1.18) | 0.498 | 0.90 (0.68 to 1.19) | 0.465 | 0.92 (0.51 to 1.65) | 0.779 | 0.65 (0.36 to 1.15) | 0.136 | 0.92 (0.67 to 1.26) | 0.599 | 1.40 (0.77 to 2.54) | 0.272 |
| High social support v low social support (3-point scale) | 0.57 (0.52 to 0.62) | 0.000 | 0.59 (0.53 to 0.65) | 0.000 | 0.54 (0.46 to 0.62) | 0.000 | 0.55 (0.47 to 0.64) | 0.000 | 0.60 (0.54 to 0.68) | 0.000 | 0.50 (0.41 to 0.62) | 0.000 |
| Moderate social support v low social support (3-point scale) | 0.88 (0.81 to 0.95) | 0.001 | 0.87 (0.79 to 0.97) | 0.008 | 0.90 (0.78 to 1.03) | 0.120 | 0.90 (0.77 to 1.05) | 0.180 | 0.89 (0.80 to 0.99) | 0.032 | 0.80 (0.65 to 0.97) | 0.027 |
| Spending more than 2 hours a day reading COVID-19 news or information^d^ | 1.16 (1.08 to 1.25) | 0.000 | 1.12 (1.02 to 1.22) | 0.019 | 1.25 (1.10 to 1.42) | 0.001 | 1.03 (0.88 to 1.21) | 0.679 | 1.24 (1.12 to 1.37) | 0.000 | 1.09 (0.92 to 1.28) | 0.313 |
| Having a balcony, terrace, or garden^d^ | 0.88 (0.79 to 0.98) | 0.018 | 0.91 (0.80 to 1.04) | 0.154 | 0.82 (0.68 to 1.00) | 0.048 | 0.94 (0.79 to 1.13) | 0.520 | 0.89 (0.77 to 1.03) | 0.105 | 0.73 (0.54 to 0.99) | 0.046 |
| Weeks in confinement (9+ versus <9) | 0.87 (0.76 to 0.99) | 0.036 | 0.84 (0.71 to 0.98) | 0.031 | 0.97 (0.76 to 1.24) | 0.794 | 0.96 (0.74 to 1.25) | 0.781 | 0.83 (0.69 to 1.00) | 0.050 | 0.76 (0.55 to 1.05) | 0.095 |
| Number of people in house (5+ versus <5) | 0.91 (0.83 to 0.99) | 0.036 | 0.91 (0.81 to 1.02) | 0.094 | 0.91 (0.77 to 1.07) | 0.252 | 0.91 (0.78 to 1.07) | 0.279 | 0.90 (0.79 to 1.01) | 0.072 | 0.83 (0.57 to 1.19) | 0.304 |
| Number of rooms in house (4+ versus <4) | 1.08 (1.02 to 1.14) | 0.006 | 1.08 (1.01 to 1.15) | 0.031 | 1.09 (0.98 to 1.20) | 0.101 | 1.04 (0.94 to 1.15) | 0.473 | 1.13 (1.05 to 1.22) | 0.001 | 0.95 (0.82 to 1.09) | 0.480 |
| Being a woman versus not | 1.79 (1.68 to 1.90) | 0.000 |  |  |  |  | 1.87 (1.66 to 2.11) | 0.000 | 1.69 (1.56 to 1.84) | 0.000 | 2.02 (1.75 to 2.34) | 0.000 |
| Primary and secondary education versus postgraduate education (3-point scale) | 1.36 (1.26 to 1.47) | 0.000 | 1.43 (1.30 to 1.57) | 0.000 | 1.23 (1.07 to 1.42) | 0.003 | 1.32 (1.15 to 1.52) | 0.000 | 1.30 (1.17 to 1.45) | 0.000 | 1.65 (1.30 to 2.08) | 0.000 |
| University education versus postgraduate education (3-point scale) | 1.31 (1.23 to 1.40) | 0.000 | 1.37 (1.27 to 1.48) | 0.000 | 1.21 (1.08 to 1.35) | 0.001 | 1.31 (1.16 to 1.49) | 0.000 | 1.32 (1.21 to 1.44) | 0.000 | 1.21 (1.04 to 1.40) | 0.011 |
| Aged 65+ versus aged 15-44 years (3-point scale) | 0.50 (0.44 to 0.58) | 0.000 | 0.55 (0.46 to 0.67) | 0.000 | 0.46 (0.36 to 0.57) | 0.000 |  |  |  |  |  |  |
| Aged 45-64 versus aged 15-44 years (3-point scale) | 0.63 (0.59 to 0.68) | 0.000 | 0.63 (0.58 to 0.69) | 0.000 | 0.64 (0.56 to 0.72) | 0.000 |  |  |  |  |  |  |
| Own job future is very likely to get worse^e^ | 1.54 (1.41 to 1.68) | 0.000 | 1.47 (1.32 to 1.64) | 0.000 | 1.70 (1.45 to 1.98) | 0.000 | 1.58 (1.36 to 1.84) | 0.000 | 1.56 (1.38 to 1.76) | 0.000 | 1.38 (1.03 to 1.84) | 0.030 |
| Worried about economic consequences of COVID-19^f^ | 0.88 (0.82 to 0.94) | 0.000 | 0.88 (0.81 to 0.96) | 0.003 | 0.87 (0.76 to 0.99) | 0.037 | 0.77 (0.67 to 0.89) | 0.001 | 0.92 (0.84 to 1.01) | 0.086 | 0.92 (0.77 to 1.11) | 0.390 |
| Being a health worker versus not | 1.11 (0.94 to 1.30) | 0.211 | 1.04 (0.86 to 1.26) | 0.676 | 1.33 (0.95 to 1.85) | 0.093 | 1.16 (0.87 to 1.55) | 0.321 | 1.10 (0.90 to 1.35) | 0.349 | 0.72 (0.32 to 1.61) | 0.419 |
| Being another front-line worker (excluding health worker) versus not | 0.91 (0.78 to 1.04) | 0.172 | 0.89 (0.76 to 1.06) | 0.196 | 0.97 (0.73 to 1.28) | 0.816 | 0.92 (0.71 to 1.20) | 0.548 | 0.88 (0.73 to 1.05) | 0.141 | 1.31 (0.55 to 3.13) | 0.545 |
| Being another worker (excluding health worker and other frontline worker) versus not | 1.18 (1.05 to 1.34) | 0.008 | 1.15 (1.00 to 1.33) | 0.058 | 1.32 (1.02 to 1.72) | 0.037 | 1.25 (0.99 to 1.58) | 0.063 | 1.14 (0.97 to 1.33) | 0.107 | 1.01 (0.59 to 1.71) | 0.977 |
| Being on sick leave versus not | 1.20 (0.99 to 1.46) | 0.067 | 1.21 (0.96 to 1.52) | 0.099 | 1.17 (0.80 to 1.72) | 0.419 | 0.87 (0.59 to 1.29) | 0.495 | 1.28 (1.02 to 1.62) | 0.034 | 1.32 (0.56 to 3.11) | 0.518 |
| Being unemployed versus not | 1.06 (0.93 to 1.22) | 0.382 | 0.97 (0.83 to 1.14) | 0.753 | 1.34 (1.00 to 1.80) | 0.047 | 1.14 (0.89 to 1.47) | 0.302 | 0.95 (0.79 to 1.13) | 0.531 | 1.09 (0.46 to 2.56) | 0.845 |
| Being a student versus not | 2.23 (1.73 to 2.88) | 0.000 | 2.31 (1.70 to 3.16) | 0.000 | 2.10 (1.32 to 3.35) | 0.002 | 1.99 (1.44 to 2.76) | 0.000 | 2.00 (0.19 to 20.93) | 0.561 | 4E+010 (0.00 to .) | 1.000 |
| Being retired versus not | 0.97 (0.82 to 1.13) | 0.678 | 0.89 (0.73 to 1.09) | 0.255 | 1.14 (0.84 to 1.54) | 0.388 | 0.78 (0.07 to 8.96) | 0.840 | 0.84 (0.69 to 1.03) | 0.086 | 1.25 (0.81 to 1.93) | 0.314 |

^a^Worry a lot (score 4 on a 4-point Likert scale from 1, not worried to 4, worried a lot) versus not score 4

^b^Almost every day (score 4 on a 4-point Likert scale from 1, not any day to 4, almost every day) versus not score 4

^c^Not any day (score 1 on a 4-point Likert scale from 1, not any day to 4, almost every day) versus not score 1

^d^Present versus absent

^e^Own job future very likely to get worse (score 5 on a 5-point Likert scale from 1, very likely to get better to 5, very likely to get worse) versus not score 5

^f^Very worried (score 10 on a 10-point Likert scale from 1, not at all worried to 10, very worried) versus not score 10

**Supplement Table 5** Odds ratios (95% confidence intervals) and probability (p) values for associations between risk and protective variables and reported depression, moderately severe depression, severe depression, anxiety and severe anxiety

|  | **Depression** | | **Moderately severe depression** | | **Severe depression** | | **Anxiety** | | **Severe anxiety** | |
| --- | --- | --- | --- | --- | --- | --- | --- | --- | --- | --- |
| **Variables** | **OR (95% CI)** | **P value** | **OR (95% CI)** | **P value** | **OR (95% CI)** | **P value** | **OR (95% CI)** | **P value** | **OR (95% CI)** | **P value** |
| Worry about being infected by Covid-19 (onself or family member)^a^ | 1.17  (1.10 to 1.24) | 0.000 | 1.08  (0.97 to 1.20) | 0.137 | 1.03  (0.87 to 1.21) | 0.735 | 1.49  (1.40 to 1.58) | 0.000 | 1.58  (1.44 to 1.73) | 0.000 |
| Worry about being alone and not being able to take care of oneself ^a^ | 1.17  (1.07 to 1.28) | 0.000 | 1.11  (0.97 to 1.27) | 0.147 | 1.44  (1.20 to 1.73) | 0.000 | 1.22  (1.12 to 1.32) | 0.000 | 1.25  (1.12 to 1.40) | 0.000 |
| Worry about family members who are alone ^a^ | 1.17  (1.10 to 1.25) | 0.000 | 1.30  (1.17 to 1.44) | 0.000 | 1.05  (0.89 to 1.23) | 0.550 | 1.22  (1.15 to 1.29) | 0.000 | 1.20  (1.10 to 1.30) | 0.000 |
| Worry about having a health emergency and not receiving any care (oneself or family member) ^a^ | 1.09  (1.02 to 1.16) | 0.007 | 1.12  (1.00 to 1.24) | 0.043 | 1.07  (0.90 to 1.27) | 0.426 | 1.14  (1.07 to 1.21) | 0.000 | 1.20  (1.09 to 1.31) | 0.000 |
| Worry about job loss ^a^ | 0.96  (0.89 to 1.04) | 0.300 | 0.95  (0.84 to 1.08) | 0.423 | 0.87  (0.72 to 1.04) | 0.117 | 1.01  (0.94 to 1.09) | 0.762 | 0.88  (0.79 to 0.98) | 0.016 |
| Worry about economic difficulties to buy food, pay rent, electricity etc. ^a^ | 1.13  (1.05 to 1.23) | 0.002 | 1.07  (0.94 to 1.21) | 0.305 | 1.28  (1.07 to 1.53) | 0.008 | 1.12  (1.04 to 1.21) | 0.003 | 1.07  (0.96 to 1.19) | 0.231 |
| Worry about loss of an academic year or the educational future of own children ^a^ | 0.96  (0.89 to 1.04) | 0.362 | 0.95  (0.83 to 1.08) | 0.434 | 1.00  (0.84 to 1.20) | 0.969 | 0.94  (0.87 to 1.02) | 0.131 | 0.89  (0.80 to 0.99) | 0.036 |
| Worry about inability to combine having to work from home while taking care of family (children, dependent persons, etc) ^a^ | 1.10  (1.01 to 1.19) | 0.035 | 1.14  (0.99 to 1.31) | 0.061 | 1.29  (1.06 to 1.57) | 0.011 | 1.30  (1.20 to 1.41) | 0.000 | 1.37  (1.23 to 1.53) | 0.000 |
| Worry about children being anxious, children not knowing what to do, leading to tensions and bad behaviour ^a^ | 1.45  (1.33 to 1.58) | 0.000 | 1.37  (1.19 to 1.57) | 0.000 | 1.47  (1.21 to 1.78) | 0.000 | 1.62  (1.49 to 1.76) | 0.000 | 1.48  (1.32 to 1.65) | 0.000 |
| Worry about not being able to go out of the house nor visit loved ones ^a^ | 1.39  (1.31 to 1.48) | 0.000 | 1.28  (1.15 to 1.42) | 0.000 | 1.45  (1.23 to 1.70) | 0.000 | 1.38  (1.30 to 1.46) | 0.000 | 1.35  (1.23 to 1.47) | 0.000 |
| Worry about the uncertainty of when and how normality will return ^a^ | 1.59  (1.49 to 1.70) | 0.000 | 1.56  (1.40 to 1.74) | 0.000 | 1.87  (1.57 to 2.23) | 0.000 | 1.89  (1.78 to 2.01) | 0.000 | 2.05  (1.87 to 2.25) | 0.000 |
| Worry about the impact the situation will have in the community (e.g., own neighborhood) ^a^ | 1.03  (0.96 to 1.10) | 0.388 | 1.07  (0.97 to 1.19) | 0.185 | 1.06  (0.91 to 1.25) | 0.459 | 1.07  (1.00 to 1.14) | 0.036 | 1.10  (1.01 to 1.20) | 0.034 |
| Worry about problems living at home ^a^ | 1.80  (1.63 to 1.99) | 0.000 | 1.57  (1.35 to 1.82) | 0.000 | 2.58  (2.14 to 3.11) | 0.000 | 2.05  (1.86 to 2.27) | 0.000 | 2.44  (2.17 to 2.75) | 0.000 |
| Worry about violence at home^a^ | 0.96  (0.83 to 1.11) | 0.541 | 0.83  (0.66 to 1.03) | 0.087 | 0.98  (0.75 to 1.28) | 0.894 | 0.88  (0.76 to 1.01) | 0.072 | 0.92  (0.77 to 1.10) | 0.370 |
| Follow a routine^b^ | 0.58  (0.55 to 0.62) | 0.000 | 0.61  (0.55 to 0.67) | 0.000 | 0.52  (0.44 to 0.61) | 0.000 | 0.80  (0.75 to 0.85) | 0.000 | 0.81  (0.74 to 0.88) | 0.000 |
| Talk with family or friends via phone or videoconference^b^ | 0.90  (0.85 to 0.96) | 0.001 | 0.81  (0.73 to 0.89) | 0.000 | 0.72  (0.62 to 0.83) | 0.000 | 0.92  (0.86 to 0.97) | 0.004 | 0.96  (0.88 to 1.05) | 0.368 |
| Watch news about Covid-19 very often (television, social media, etc)^b^ | 1.04  (0.98 to 1.11) | 0.183 | 1.06  (0.95 to 1.17) | 0.300 | 1.04  (0.89 to 1.21) | 0.643 | 1.12  (1.05 to 1.19) | 0.000 | 1.11  (1.02 to 1.21) | 0.016 |
| Take the opportunity to do housework (e.g., DIY projects, cooking, etc)^b^ | 0.80  (0.75 to 0.86) | 0.000 | 0.82  (0.73 to 0.92) | 0.001 | 0.69  (0.57 to 0.83) | 0.000 | 0.89  (0.83 to 0.95) | 0.000 | 0.83  (0.75 to 0.91) | 0.000 |
| Spend time outdoors (patio, balcony) or looking outside (street, sky, etc.)^b^ | 0.96  (0.91 to 1.03) | 0.261 | 0.97  (0.88 to 1.08) | 0.612 | 0.74  (0.63 to 0.87) | 0.000 | 0.95  (0.90 to 1.01) | 0.094 | 0.97  (0.89 to 1.06) | 0.461 |
| Undertake relaxing activities (e.g., listen to music)^b^ | 0.78  (0.73 to 0.84) | 0.000 | 0.71  (0.63 to 0.81) | 0.000 | 0.73  (0.60 to 0.89) | 0.002 | 0.72  (0.68 to 0.77) | 0.000 | 0.73  (0.66 to 0.81) | 0.000 |
| Dedicate time to oneself^b^ | 0.60  (0.54 to 0.67) | 0.000 | 0.52  (0.42 to 0.64) | 0.000 | 0.49  (0.34 to 0.70) | 0.000 | 0.69  (0.63 to 0.76) | 0.000 | 0.61  (0.52 to 0.71) | 0.000 |
| Spend more time doing activities with family^b^ | 0.70  (0.65 to 0.76) | 0.000 | 0.66  (0.58 to 0.76) | 0.000 | 0.63  (0.51 to 0.78) | 0.000 | 0.73  (0.68 to 0.78) | 0.000 | 0.66  (0.60 to 0.74) | 0.000 |
| Take the opportunity to learn new things (new studies, hobbies, etc) ^b^ | 0.88  (0.78 to 0.99) | 0.032 | 0.93  (0.75 to 1.15) | 0.515 | 1.01  (0.71 to 1.43) | 0.969 | 0.84  (0.76 to 0.94) | 0.003 | 0.87  (0.73 to 1.03) | 0.111 |
| Take the opportunity to advance paperwork or other delayed activities ^b^ | 0.94  (0.86 to 1.03) | 0.167 | 0.87  (0.75 to 1.01) | 0.075 | 0.73  (0.56 to 0.95) | 0.021 | 1.05  (0.96 to 1.13) | 0.284 | 1.07  (0.95 to 1.21) | 0.258 |
| Does not eat more to cope with situation^c^ | 0.63  (0.59 to 0.67) | 0.000 | 0.66  (0.59 to 0.74) | 0.000 | 0.82  (0.69 to 0.97) | 0.021 | 0.88  (0.83 to 0.93) | 0.000 | 0.93  (0.85 to 1.02) | 0.126 |
| Does not drink more alcohol to cope with the situation^c^ | 0.78  (0.73 to 0.83) | 0.000 | 0.81  (0.73 to 0.90) | 0.000 | 0.70  (0.60 to 0.82) | 0.000 | 0.83  (0.78 to 0.88) | 0.000 | 0.83  (0.76 to 0.91) | 0.000 |
| Has a job that increases risk of COVID-19^d^ | 1.03  (0.93 to 1.13) | 0.596 | 1.14  (0.97 to 1.32) | 0.104 | 1.10  (0.87 to 1.39) | 0.415 | 1.10  (1.01 to 1.20) | 0.036 | 1.18  (1.04 to 1.34) | 0.012 |
| Suffer from chronic diseases that increase own risk of COVID-19 infection^d^ | 1.56  (1.44 to 1.68) | 0.000 | 1.52  (1.35 to 1.72) | 0.000 | 1.52  (1.28 to 1.82) | 0.000 | 1.36  (1.26 to 1.47) | 0.000 | 1.34  (1.20 to 1.49) | 0.000 |
| Have had COVID-19 with mild symptomatology ^d^ | 1.33  (1.21 to 1.45) | 0.000 | 1.25  (1.08 to 1.44) | 0.003 | 1.36  (1.11 to 1.68) | 0.004 | 1.24  (1.14 to 1.36) | 0.000 | 1.32  (1.17 to 1.50) | 0.000 |
| Have had COVID-19 with severe symptomatology ^d^ | 1.55  (1.18 to 2.03) | 0.001 | 1.48  (0.99 to 2.21) | 0.059 | 3.00  (1.86 to 4.84) | 0.000 | 1.42  (1.09 to 1.85) | 0.009 | 1.55  (1.11 to 2.18) | 0.011 |
| Have family or friends with risk factors for COVID-19 (e.g., older age, previous diseases) ^d^ | 1.08  (1.02 to 1.15) | 0.012 | 1.15  (1.04 to 1.28) | 0.005 | 1.13  (0.97 to 1.32) | 0.117 | 1.13  (1.07 to 1.20) | 0.000 | 1.10  (1.01 to 1.20) | 0.034 |
| Have family or friends with COVID-19 but not hospitalised ^d^ | 1.01  (0.95 to 1.08) | 0.738 | 1.01  (0.90 to 1.12) | 0.902 | 0.94  (0.80 to 1.12) | 0.495 | 0.99  (0.93 to 1.06) | 0.833 | 0.99  (0.90 to 1.08) | 0.773 |
| Have family or friends with COVID-19 hospitalised ^d^ | 0.99  (0.92 to 1.06) | 0.752 | 1.00  (0.89 to 1.13) | 0.958 | 1.04  (0.87 to 1.25) | 0.672 | 1.05  (0.98 to 1.12) | 0.202 | 1.08  (0.97 to 1.19) | 0.162 |
| Have family or a close friend who has died because of COVID-19 ^d^ | 1.12  (1.04 to 1.20) | 0.004 | 1.02  (0.90 to 1.16) | 0.713 | 1.24  (1.03 to 1.49) | 0.022 | 1.10  (1.03 to 1.19) | 0.007 | 1.10  (0.99 to 1.22) | 0.089 |
| Have children and they take up half of my time or less^d^ | 0.76  (0.70 to 0.83) | 0.000 | 0.68  (0.59 to 0.79) | 0.000 | 0.66  (0.53 to 0.83) | 0.000 | 1.00  (0.93 to 1.09) | 0.902 | 0.86  (0.76 to 0.97) | 0.011 |
| Have children and they take up most of my time^d^ | 0.82  (0.75 to 0.90) | 0.000 | 0.71  (0.61 to 0.83) | 0.000 | 0.70  (0.55 to 0.88) | 0.002 | 1.10  (1.01 to 1.20) | 0.036 | 1.08  (0.96 to 1.23) | 0.195 |
| Be responsible for a dependent person/s and they take up half of my time or less^d^ | 1.16  (1.02 to 1.32) | 0.020 | 1.00  (0.81 to 1.23) | 0.997 | 1.08  (0.80 to 1.44) | 0.627 | 1.15  (1.02 to 1.30) | 0.023 | 1.18  (0.99 to 1.40) | 0.058 |
| Be responsible for a dependent person/s and they take up most of the time^d^ | 1.27  (1.01 to 1.60) | 0.040 | 0.95  (0.65 to 1.38) | 0.770 | 1.62  (1.04 to 2.51) | 0.034 | 1.61  (1.30 to 2.00) | 0.000 | 1.81  (1.37 to 2.40) | 0.000 |
| High social support v low social support (3-point scale) | 0.54  (0.50 to 0.59) | 0.000 | 0.54  (0.47 to 0.62) | 0.000 | 0.34  (0.28 to 0.43) | 0.000 | 0.65  (0.60 to 0.71) | 0.000 | 0.50  (0.44 to 0.56) | 0.000 |
| Moderate social support v low social support (3-point scale) | 0.74  (0.69 to 0.79) | 0.000 | 0.72  (0.64 to 0.80) | 0.000 | 0.49  (0.42 to 0.57) | 0.000 | 0.80  (0.75 to 0.86) | 0.000 | 0.62  (0.56 to 0.68) | 0.000 |
| Spending more than 2 hours a day reading COVID-19 news or information^d^ | 1.18  (1.09 to 1.27) | 0.000 | 1.03  (0.91 to 1.16) | 0.638 | 1.27  (1.07 to 1.50) | 0.007 | 1.32  (1.23 to 1.41) | 0.000 | 1.24  (1.12 to 1.37) | 0.000 |
| Having a balcony, terrace, or garden^d^ | 0.95  (0.87 to 1.05) | 0.318 | 0.96  (0.83 to 1.12) | 0.623 | 0.87  (0.71 to 1.07) | 0.190 | 0.88  (0.81 to 0.97) | 0.007 | 0.89  (0.78 to 1.01) | 0.070 |
| Weeks in confinement (9+ versus <9) | 1.13  (1.00 to 1.29) | 0.058 | 1.25  (1.02 to 1.53) | 0.034 | 1.68  (1.29 to 2.20) | 0.000 | 1.06  (0.93 to 1.20) | 0.401 | 1.18  (0.99 to 1.41) | 0.068 |
| Number of people in house (5+ versus <5) | 1.01  (0.92 to 1.11) | 0.837 | 1.01  (0.87 to 1.18) | 0.892 | 0.91  (0.72 to 1.15) | 0.445 | 1.04  (0.95 to 1.14) | 0.399 | 0.98  (0.86 to 1.12) | 0.800 |
| Number of rooms in house (4+ versus <4) | 1.07  (1.01 to 1.13) | 0.033 | 1.02  (0.92 to 1.12) | 0.757 | 1.11  (0.95 to 1.29) | 0.183 | 1.05  (0.99 to 1.11) | 0.098 | 1.03  (0.95 to 1.12) | 0.459 |
| Being a woman versus not | 1.64  (1.52 to 1.76) | 0.000 | 1.56  (1.38 to 1.76) | 0.000 | 1.59  (1.32 to 1.92) | 0.000 | 1.57  (1.47 to 1.69) | 0.000 | 1.53  (1.38 to 1.70) | 0.000 |
| Primary and secondary education versus postgraduate education (3-point scale) | 1.09  (1.00 to 1.18) | 0.051 | 0.91  (0.79 to 1.04) | 0.166 | 0.88  (0.71 to 1.10) | 0.256 | 0.93  (0.86 to 1.00) | 0.065 | 0.84  (0.75 to 0.95) | 0.005 |
| University education versus postgraduate education (3-point scale) | 1.02  (0.95 to 1.09) | 0.534 | 0.90  (0.81 to 1.01) | 0.073 | 0.81  (0.68 to 0.97) | 0.018 | 0.93  (0.87 to 0.99) | 0.021 | 0.91  (0.83 to 1.00) | 0.060 |
| Aged 65+ versus aged 15-44 years (3-point scale) | 0.45  (0.37 to 0.54) | 0.000 | 0.51  (0.36 to 0.71) | 0.000 | 0.52  (0.31 to 0.88) | 0.016 | 0.61  (0.51 to 0.72) | 0.000 | 0.45  (0.34 to 0.59) | 0.000 |
| Aged 45-64 versus aged 15-44 years (3-point scale) | 0.63  (0.59 to 0.67) | 0.000 | 0.54  (0.48 to 0.60) | 0.000 | 0.68  (0.58 to 0.81) | 0.000 | 0.59  (0.55 to 0.63) | 0.000 | 0.53  (0.48 to 0.58) | 0.000 |
| Own job future is very likely to get worse^e^ | 1.60  (1.49 to 1.72) | 0.000 | 1.47  (1.31 to 1.65) | 0.000 | 1.98  (1.68 to 2.33) | 0.000 | 1.49  (1.39 to 1.60) | 0.000 | 1.54  (1.40 to 1.70) | 0.000 |
| Worried about economic consequences of COVID-19^f^ | 1.31  (1.23 to 1.41) | 0.000 | 1.37  (1.23 to 1.53) | 0.000 | 2.04  (1.74 to 2.39) | 0.000 | 1.46  (1.37 to 1.56) | 0.000 | 1.81  (1.66 to 1.98) | 0.000 |
| Being a health worker versus not | 1.05  (0.89 to 1.23) | 0.550 | 0.81  (0.63 to 1.06) | 0.121 | 0.63  (0.42 to 0.93) | 0.021 | 1.38  (1.18 to 1.62) | 0.000 | 1.01  (0.81 to 1.26) | 0.954 |
| Being another front-line worker (excluding health worker) versus not | 0.83  (0.71 to 0.96) | 0.014 | 0.78  (0.62 to 1.00) | 0.049 | 0.68  (0.47 to 0.98) | 0.037 | 1.03  (0.90 to 1.19) | 0.640 | 0.87  (0.71 to 1.06) | 0.171 |
| Being another worker (excluding health worker and other frontline worker) versus not | 0.91  (0.81 to 1.03) | 0.152 | 0.85  (0.69 to 1.04) | 0.112 | 0.73  (0.55 to 0.98) | 0.036 | 1.07  (0.94 to 1.21) | 0.298 | 0.87  (0.73 to 1.03) | 0.103 |
| Being on sick leave versus not | 1.50  (1.26 to 1.78) | 0.000 | 1.54  (1.19 to 2.00) | 0.001 | 2.07  (1.47 to 2.90) | 0.000 | 1.28  (1.08 to 1.53) | 0.004 | 1.49  (1.19 to 1.88) | 0.001 |
| Being unemployed versus not | 1.03  (0.90 to 1.17) | 0.701 | 0.94  (0.76 to 1.16) | 0.573 | 0.96  (0.72 to 1.29) | 0.791 | 1.11  (0.97 to 1.26) | 0.131 | 0.92  (0.76 to 1.10) | 0.362 |
| Being a student versus not | 2.13  (1.77 to 2.56) | 0.000 | 1.68  (1.28 to 2.22) | 0.000 | 1.69  (1.14 to 2.52) | 0.009 | 1.70  (1.41 to 2.05) | 0.000 | 1.55  (1.20 to 2.01) | 0.001 |
| Being retired versus not | 0.81  (0.67 to 0.98) | 0.032 | 0.66  (0.47 to 0.93) | 0.019 | 0.57  (0.34 to 0.98) | 0.043 | 0.79  (0.65 to 0.95) | 0.013 | 0.68  (0.51 to 0.92) | 0.011 |

**Supplement Table 6a** Path analysis. Direct path excluding mental health conditions in model (**BLUE**) (Factors independent variables) to substance use outcomes (dependent variables).

|  | |  | **Direct path: Factor ⇒ outcome** | | | | | | |
| --- | --- | --- | --- | --- | --- | --- | --- | --- | --- |
|  | **Mental health condition** |  |  | **AUDIT-C positive (5+)** | **Smokes** | **Smoked more cigarettes during confinement** | **Non-prescription hypnosedatives** | **Prescription hypnosedatives** | **Cannabis** |
|  |  |  | Worry about the uncertainty of when and how we will retake normality | 1.12 (1.03 to 1.21) | **1.30 (1.24 to 1.37)** | **1.26 (1.15 to 1.37)** | 1.25 (1.16 to 1.36) | **1.38 (1.30 to 1.46)** | 1.03 (0.90 to 1.17) |
|  |  |  | Worry about problems living at home | **1.31 (1.14 to 1.51)** | 1.07 (0.98 to 1.18) | 1.17 (1.00 to 1.36) | **1.60 (1.41 to 1.81)** | **1.39 (1.26 to 1.53)** | 1.15 (0.93 to 1.43) |
|  |  |  | Suffer from chronic diseases that increase own risk of COVID-19 infection | 0.95 (0.84 to 1.06) | 1.00 (0.93 to 1.07) | 1.15 (1.02 to 1.31) | 1.11 (0.99 to 1.23) | **1.97 (1.84 to 2.11)** | 1.09 (0.91 to 1.32) |
|  |  |  | Worry about uncertain future of own job | 1.25 (1.13 to 1.39) | 1.21 (1.13 to 1.29) | 1.19 (1.07 to 1.32) | **1.31 (1.19 to 1.44)** | 1.15 (1.07 to 1.24) | **1.49 (1.29 to 1.72)** |
|  | **Depression** |  |  |  |  |  |  |  |  |
|  |  |  | Following a routine | 0.83 (0.77 to 0.91) | 0.81 (0.76 to 0.85) | 0.78 (0.71 to 0.85) | **0.73 (0.67 to 0.79)** | **0.63 (0.60 to 0.67)** | **0.68 (0.59 to 0.77)** |
|  |  |  | Dedicating time to oneself | 1.00 (0.89 to 1.12) | 0.92 (0.85 to 0.99) | **0.67 (0.59 to 0.76)** | **0.79 (0.70 to 0.89)** | 0.82 (0.75 to 0.89) | 1.01 (0.84 to 1.22) |
|  |  |  | Not eating more to cope with the situation | **0.67 (0.62 to 0.74)** | 0.98 (0.93 to 1.03) | **0.60 (0.54 to 0.66)** | 0.87 (0.80 to 0.95) | 0.95 (0.90 to 1.01) | 0.89 (0.78 to 1.02) |
|  |  |  | Having a high level of social support | 0.92 (0.83 to 1.02) | 0.96 (0.90 to 1.03) | **0.79 (0.70 to 0.88)** | **0.77 (0.70 to 0.84)** | 0.81 (0.76 to 0.87) | 1.06 (0.90 to 1.24) |
|  |  |  |  |  |  |  |  |  |  |
|  |  |  | Worry about anxious children who do not know what to do, leading to tensions and bad child behaviour | 1.07 (0.94 to 1.21) | 1.04 (0.96 to 1.12) | **1.37 (1.20 to 1.55)** | 1.09 (0.97 to 1.22) | 1.23 (1.13 to 1.33) | **0.57 (0.45 to 0.71)** |
|  |  |  | Worry about the uncertainty of when and how we will retake normality | 1.17 (1.07 to 1.27) | 1.34 (1.27 to 1.41) | **1.28 (1.18 to 1.40)** | **1.31 (1.21 to 1.42)** | **1.41 (1.33 to 1.49)** | 1.14 (1.01 to 1.30) |
|  |  |  | Worry about problems living at home | **1.35 (1.16 to 1.56)** | 1.09 (0.99 to 1.20) | 1.12 (0.95 to 1.31) | **1.65 (1.45 to 1.88)** | **1.37 (1.24 to 1.52)** | **1.46 (1.17 to 1.81)** |
|  |  |  | Being responsible for dependent people that take up most of the time | 1.14 (0.80 to 1.61) | **1.83 (1.51 to 2.22)** | 1.18 (0.87 to 1.61) | 1.13 (0.82 to 1.54) | **1.59 (1.30 to 1.95)** | **1.39 (0.82 to 2.36)** |
|  | **Anxiety** |  |  |  |  |  |  |  |  |
|  |  |  | Having a high level of social support | 0.85 (0.76 to 0.95) | 1.04 (0.97 to 1.12) | **0.67 (0.59 to 0.75)** | **0.57 (0.51 to 0.64)** | **0.59 (0.55 to 0.64)** | 0.92 (0.77 to 1.09) |
|  |  |  |  |  |  |  |  |  |  |
|  |  |  | Worry about the uncertainty of when and how we will retake normality | 1.13 (1.04 to 1.23) | **1.30 (1.24 to 1.37)** | **1.26 (1.15 to 1.37)** | **1.26 (1.16 to 1.36)** | **1.39 (1.31 to 1.47)** | 1.03 (0.91 to 1.17) |
|  |  |  | Worry about problems living at home | **1.34 (1.16 to 1.54)** | 1.07 (0.98 to 1.18) | 1.17 (1.00 to 1.36) | **1.62 (1.43 to 1.83)** | **1.41 (1.28 to 1.56)** | 1.16 (0.94 to 1.44) |
|  |  |  | Worry about uncertain future of own job | **1.27 (1.15 to 1.41)** | 1.21 (1.13 to 1.29) | 1.19 (1.07 to 1.32) | **1.32 (1.20 to 1.45)** | 1.16 (1.08 to 1.24) | **1.50 (1.30 to 1.73)** |
|  | **Lack of wellbeing** |  |  |  |  |  |  |  |  |
|  |  |  | Dedicating time to oneself | 0.81 (0.74 to 0.88) | 0.80 (0.76 to 0.85) | **0.78 (0.71 to 0.85)** | **0.72 (0.66 to 0.78)** | **0.62 (0.59 to 0.66)** | **0.67 (0.59 to 0.76)** |
|  |  |  | Not eating more to cope with the situation | 0.97 (0.86 to 1.09) | 0.92 (0.85 to 0.98) | **0.67 (0.59 to 0.76)** | **0.78 (0.69 to 0.88)** | 0.81 (0.75 to 0.88) | 1.00 (0.83 to 1.21) |
|  |  |  | Having a high level of social support | 0.90 (0.80 to 1.01) | 1.10 (1.02 to 1.18) | **0.75 (0.66 to 0.84)** | **0.63 (0.56 to 0.70)** | **0.64 (0.60 to 0.70)** | 1.02 (0.86 to 1.22) |

**Supplement Table 6b** Path analysis. Indirect path (**GREEN**): Model (a) Factor (independent variables) to mental health conditions (dependent variables); Model (b) Mental health conditions (independent variables, with factors added to model) to substance use outcomes (dependent variables). Direct path (**BLUE**) (Factors independent variables, with mental health conditions added to model) to substance use outcomes (dependent variables).

| **Indirect path: Factor ⇒ Mental health condition ⇒ outcome** | |  | **Direct path: Factor ⇒ outcome** | | | | | | |
| --- | --- | --- | --- | --- | --- | --- | --- | --- | --- |
| **Factor** | **Mental health condition** |  |  | **AUDIT-C positive (5+)** | **Smokes** | **Smoked more cigarettes during confinement** | **Non-prescription hypnosedatives** | **Prescription hypnosedatives** | **Cannabis** |
| Worry about the uncertainty of when and how we will retake normality | **2.13 (2.02 to 2.25)** |  | Worry about the uncertainty of when and how we will retake normality | 1.09 (1.00 to 1.18) | **1.27 (1.21 to 1.34)** | 1.20 (1.09 to 1.31) | 1.13 (1.04 to 1.22) | 1.24 (1.17 to 1.31) | 1.00 (0.88 to 1.13) |
| Worry about problems living at home | **2.44 (2.23 to 2.67)** |  | Worry about problems living at home | **1.26 (1.09 to 1.45)** | 1.03 (0.94 to 1.14) | 1.09 (0.94 to 1.28) | **1.38 (1.22 to 1.57)** | 1.18 (1.07 to 1.31) | 1.11 (0.89 to 1.37) |
| Suffer from chronic diseases that increase own risk of COVID-19 infection | **1.76 (1.64 to 1.90)** |  | Suffer from chronic diseases that increase own risk of COVID-19 infection | 0.93 (0.83 to 1.04) | 0.98 (0.91 to 1.05) | 1.10 (0.97 to 1.25) | 1.02 (0.91 to 1.14) | 1.84 (1.72 to 1.98) | 1.07 (0.89 to 1.29) |
| Worry about uncertain future of own job | **1.74 (1.63 to 1.86)** |  | Worry about uncertain future of own job | 1.22 (1.11 to 1.36) | 1.18 (1.11 to 1.26) | 1.14 (1.03 to 1.27) | 1.20 (1.09 to 1.32) | 1.05 (0.97 to 1.13) | **1.46 (1.26 to 1.68)** |
|  | **Depression** |  |  | **1.27 (1.14 to 1.41)** | 1.22 (1.15 to 1.30) | **1.46 (1.32 to 1.62)** | **2.25 (2.06 to 2.46)** | **2.38 (2.23 to 2.55)** | **1.27 (1.10 to 1.47)** |
| Following a routine | **0.48 (0.45 to 0.51)** |  | Following a routine | 0.86 (0.79 to 0.93) | 0.82 (0.78 to 0.87) | 0.81 (0.74 to 0.89) | 0.81 (0.74 to 0.88) | **0.70 (0.66 to 0.74)** | **0.70 (0.61 to 0.79)** |
| Dedicating time to oneself | **0.50 (0.45 to 0.55)** |  | Dedicating time to oneself | 1.02 (0.91 to 1.14) | 0.93 (0.87 to 1.00) | **0.69 (0.61 to 0.79)** | 0.85 (0.75 to 0.97) | 0.88 (0.81 to 0.96) | 1.03 (0.85 to 1.24) |
| Eating more to cope with the situation | **0.60 (0.56 to 0.64)** |  | Not eating more to cope with the situation | **0.68 (0.62 to 0.75)** | 0.99 (0.94 to 1.04) | **0.61 (0.56 to 0.67)** | 0.93 (0.85 to 1.01) | 1.02 (0.96 to 1.08) | 0.91 (0.79 to 1.04) |
| Having a high level of social support | **0.48 (0.44 to 0.52)** |  | Having a high level of social support | 0.93 (0.83 to 1.05) | 1.12 (1.04 to 1.20) | **0.78 (0.69 to 0.88)** | **0.70 (0.63 to 0.78)** | **0.72 (0.67 to 0.78)** | 1.06 (0.89 to 1.27) |
|  |  |  |  |  |  |  |  |  |  |
| Worry about anxious children who do not know what to do, leading to tensions and bad child behaviour | **2.12 (1.98 to 2.28)** |  | Worry about anxious children who do not know what to do, leading to tensions and bad child behaviour | 1.03 (0.91 to 1.17) | 1.01 (0.94 to 1.10) | **1.29 (1.13 to 1.46)** | 0.93 (0.83 to 1.05) | 1.07 (0.98 to 1.16) | **0.56 (0.45 to 0.70)** |
| Worry about the uncertainty of when and how we will retake normality | **2.70 (2.56 to 2.84)** |  | Worry about the uncertainty of when and how we will retake normality | 1.12 (1.03 to 1.22) | **1.30 (1.24 to 1.37)** | 1.20 (1.10 to 1.31) | 1.08 (0.99 to 1.17) | 1.19 (1.13 to 1.27) | 1.12 (0.98 to 1.28) |
| Worry about problems living at home | **2.50 (2.28 to 2.73)** |  | Worry about problems living at home | **1.28 (1.10 to 1.49)** | 1.06 (0.96 to 1.16) | 1.05 (0.90 to 1.24) | **1.36 (1.19 to 1.55)** | 1.14 (1.03 to 1.27) | **1.42 (1.14 to 1.78)** |
| Being responsible for dependent people that take up most of the time | **1.83 (1.49 to 2.25)** |  | Being responsible for dependent people that take up most of the time | 1.11 (0.78 to 1.57) | **1.80 (1.48 to 2.19)** | 1.16 (0.85 to 1.57) | 1.00 (0.73 to 1.38) | **1.45 (1.18 to 1.78)** | **1.38 (0.81 to 2.33)** |
|  | **Anxiety** |  |  | **1.26 (1.14 to 1.39)** | 1.16 (1.09 to 1.23) | **1.45 (1.31 to 1.60)** | **2.72 (2.49 to 2.96)** | **2.55 (2.39 to 2.72)** | 1.11 (0.96 to 1.29) |
| Having a high level of social support | **0.52 (0.48 to 0.55)** |  | Having a high level of social support | 0.87 (0.78 to 0.98) | 1.06 (0.99 to 1.14) | **0.70 (0.62 to 0.79)** | **0.64 (0.58 to 0.72)** | **0.65 (0.60 to 0.70)** | 0.93 (0.78 to 1.11) |
|  |  |  |  |  |  |  |  |  |  |
| Worry about the uncertainty of when and how we will retake normality | **1.90 (1.79 to 2.01)** |  | Worry about the uncertainty of when and how we will retake normality | 1.11 (1.02 to 1.21) | **1.30 (1.23 to 1.37)** | 1.21 (1.11 to 1.32) | 1.20 (1.10 to 1.30) | **1.31 (1.23 to 1.38)** | 1.02 (0.89 to 1.16) |
| Worry about problems living at home | **2.30 (1.98 to 2.67)** |  | Worry about problems living at home | **1.32 (1.15 to 1.52)** | 1.07 (0.97 to 1.17) | 1.14 (0.98 to 1.33) | **1.56 (1.37 to 1.76)** | **1.35 (1.23 to 1.49)** | 1.15 (0.93 to 1.42) |
| Worry about uncertain future of own job | **1.46 (1.35 to 1.58)** |  | Worry about uncertain future of own job | **1.26 (1.14 to 1.40)** | 1.20 (1.13 to 1.28) | 1.17 (1.05 to 1.30) | **1.29 (1.17 to 1.42)** | 1.13 (1.05 to 1.21) | **1.49 (1.29 to 1.71)** |
|  | **Lack of wellbeing** |  |  | 1.20 (1.08 to 1.34) | 1.04 (0.98 to 1.11) | **1.54 (1.36 to 1.73)** | **2.20 (1.92 to 2.52)** | **2.20 (2.02 to 2.39)** | 1.20 (1.00 to 1.44) |
| Dedicating time to oneself | **0.46 (0.43 to 0.49)** |  | Dedicating time to oneself | 0.82 (0.76 to 0.89) | 0.81 (0.77 to 0.85) | 0.81 (0.74 to 0.88) | **0.76 (0.70 to 0.83)** | **0.66 (0.63 to 0.70)** | **0.68 (0.60 to 0.77)** |
| Eating more to cope with the situation | **0.39 (0.37 to 0.42)** |  | Not eating more to cope with the situation | 1.00 (0.89 to 1.13) | 0.92 (0.86 to 0.99) | **0.72 (0.63 to 0.82)** | 0.88 (0.78 to 0.99) | 0.92 (0.85 to 1.00) | 1.03 (0.85 to 1.25) |
| Having a high level of social support | **0.50 (0.47 to 0.55)** |  | Having a high level of social support | 0.92 (0.82 to 1.03) | 1.10 (1.02 to 1.18) | **0.78 (0.69 to 0.88)** | **0.67 (0.60 to 0.74)** | **0.69 (0.64 to 0.74)** | 1.04 (0.87 to 1.24) |


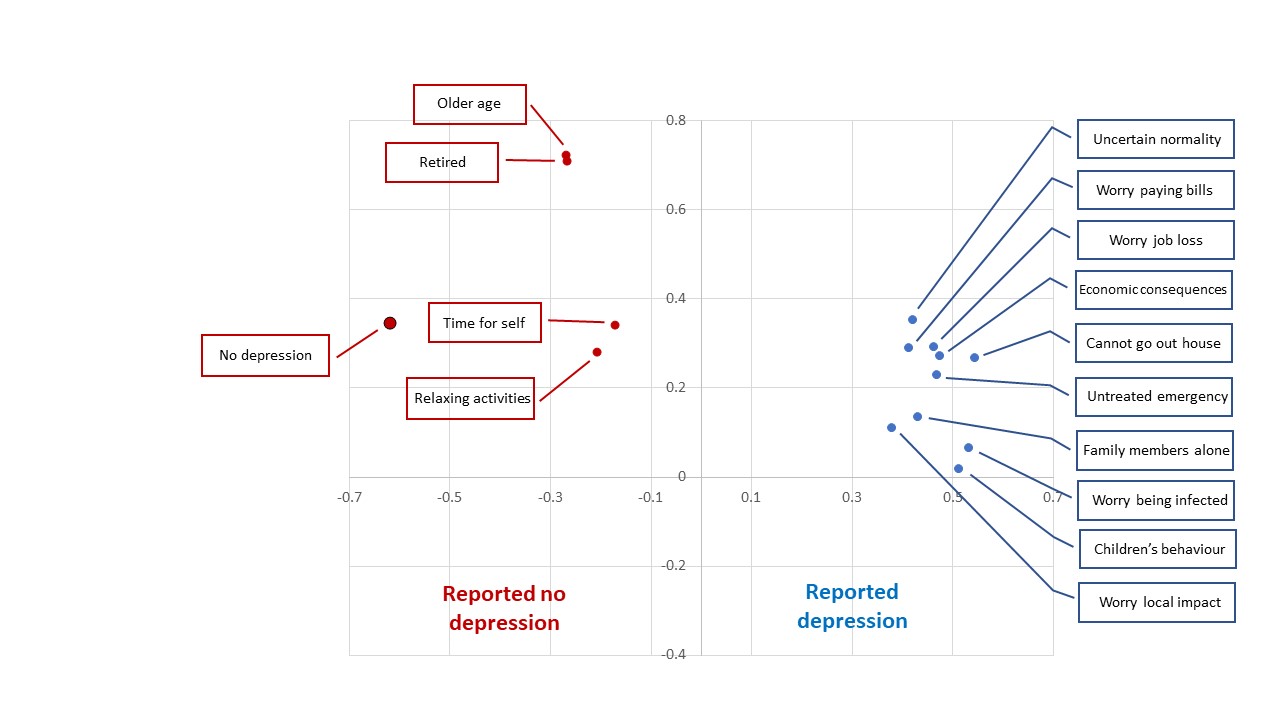


**Supplement Figure 1** Component loadings of protective and risk factors for two categories, reporting no depression and reporting depression.


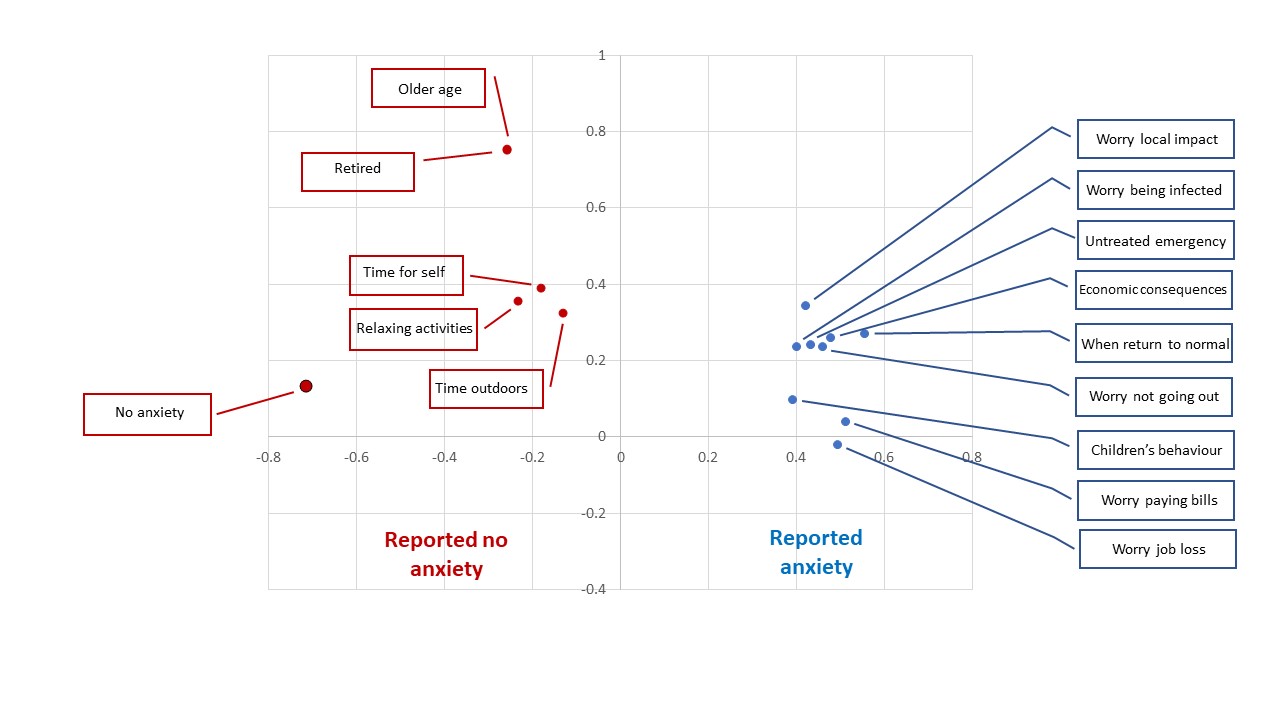


**Supplement Figure 2** Component loadings of protective and risk factors for two categories, reporting no anxiety and reporting anxiety.


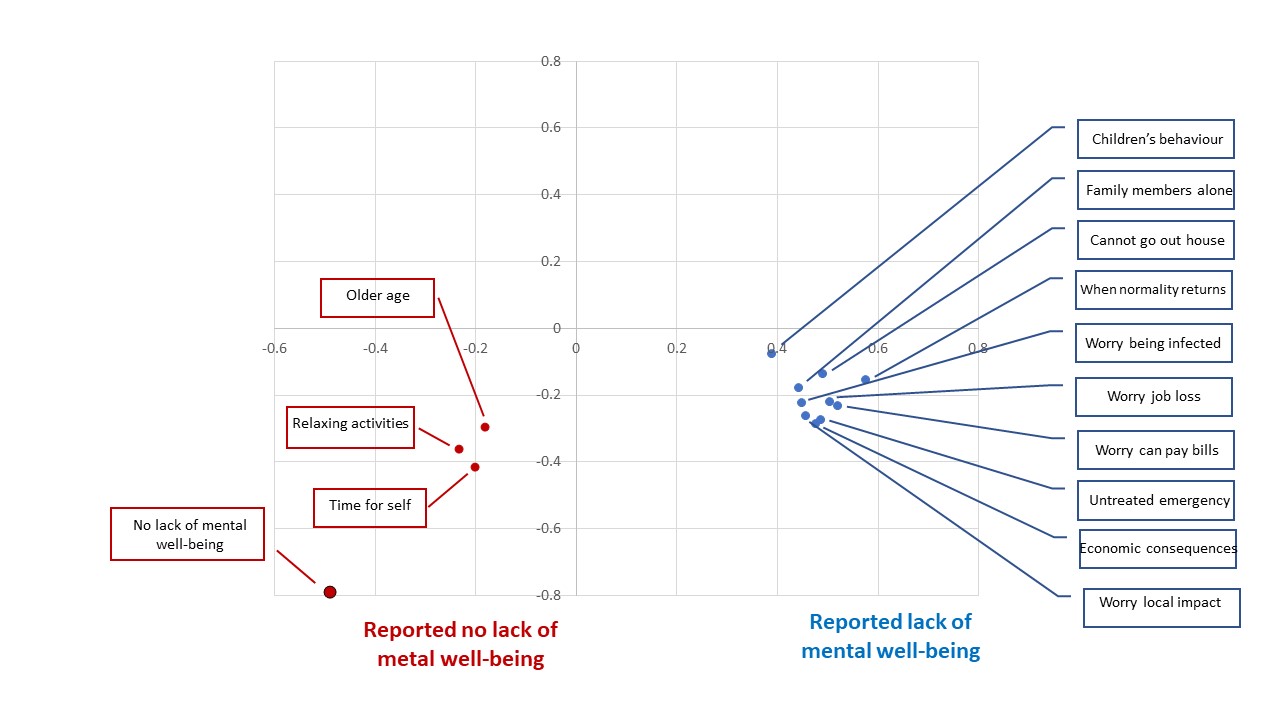


**Figure 3** Component loadings of protective and risk factors for two categories, reporting no lack of mental wellbeing and reporting lack of mental wellbeing.
